# Bacterial stress rewires diatom chronobiology and ecosystem function

**DOI:** 10.1016/j.isci.2026.117307

**Published:** 2026-08-24

**Authors:** Jiwei Chen, Hang Diao, Yixi Su, Yuan Yuan, Manish Kumar, Fan Zhang, Haisheng Zhang, Karsten Zengler, Bernhard O. Palsson, Weiqi Fu

**Affiliations:** 1 State Key Laboratory of Ocean Sensing and Ocean College, Zhejiang University, Zhoushan, Zhejiang 316021, China; 2 Department of Marine Information and Key Laboratory of Oceanographic Big Data Mining and Application of Zhejiang Province, Zhejiang University, Zhoushan, Zhejiang 316021, China; 3 Ocean Research Center of Zhoushan, Zhejiang University, Zhoushan, Zhejiang 316021, China; 4 Shu Chien-Gene Lay Department of Bioengineering, University of California, San Diego, La Jolla, CA 92093, USA; 5 Department of Pediatrics, University of California, San Diego, 9500 Gilman Drive, La Jolla, CA 92093, USA; 6 Ocean Academy, Zhejiang University, Zhoushan, Zhejiang 316021, China; 7 Department of Bioengineering, University of California, San Diego, 9500 Gilman Drive, La Jolla, CA 92093, USA; 8 Center for Microbiome Innovation, University of California, San Diego, 9500 Gilman Drive, La Jolla, CA 92093, USA; 9 Program in Materials Science and Engineering, University of California, San Diego, 9500 Gilman Drive, La Jolla, CA 92093, USA; 10 Bioinformatics and Systems Biology Program, University of California, San Diego, 9500 Gilman Drive, La Jolla, CA 92093, USA; 11 Center for Systems Biology and Faculty of Industrial Engineering, School of Engineering and Natural Sciences, University of Iceland, 101 Reykjavík, Iceland

**Keywords:** algae-bacteria co-culture, diatoms, deep learning, circadian rhythms, blue carbon

## Abstract

Diatoms are vital primary producers in marine ecosystems and play a key role in blue carbon sequestration. Although their circadian rhythms have been studied in isolation, how these rhythms are modulated by co-occurring bacteria remains unknown. Using a defined co-culture of the diatom *Phaeodactylum tricornutum* with the marine bacterium *Aliivibrio fischeri*, we observed time-dependent physiological and transcriptomic changes in *P. tricornutum*, including a 16.3% reduction in rhythmic genes with prolonged culture time. Genome-scale metabolic modeling suggested a biphasic response, with predicted biomass flux increasing by 81% at the early co-culture stage but decreasing by 87% during prolonged co-culture. Deconvolution of the transcriptome via AI-driven independent component analysis identified gene modules associated with silica transport and senescence-related responses under co-culture conditions. Together, these findings establish a systems-level framework that links interspecies interactions between diatoms and bacteria, providing mechanistic insights into how microbial associations influence phytoplankton chronobiology and rhythmic regulation.

## Highlights

• Bacterial co-culture reshapes diel transcription in *P. tricornutum*
• Metabolic modeling reveals carbon reallocation under co-culture stress
• DeepCluster ICA enables robust decomposition of time-series transcriptomes
• Co-culture rewires rhythmic gene expression and metabolic fluxes

## Graphical abstract

**Figure:**
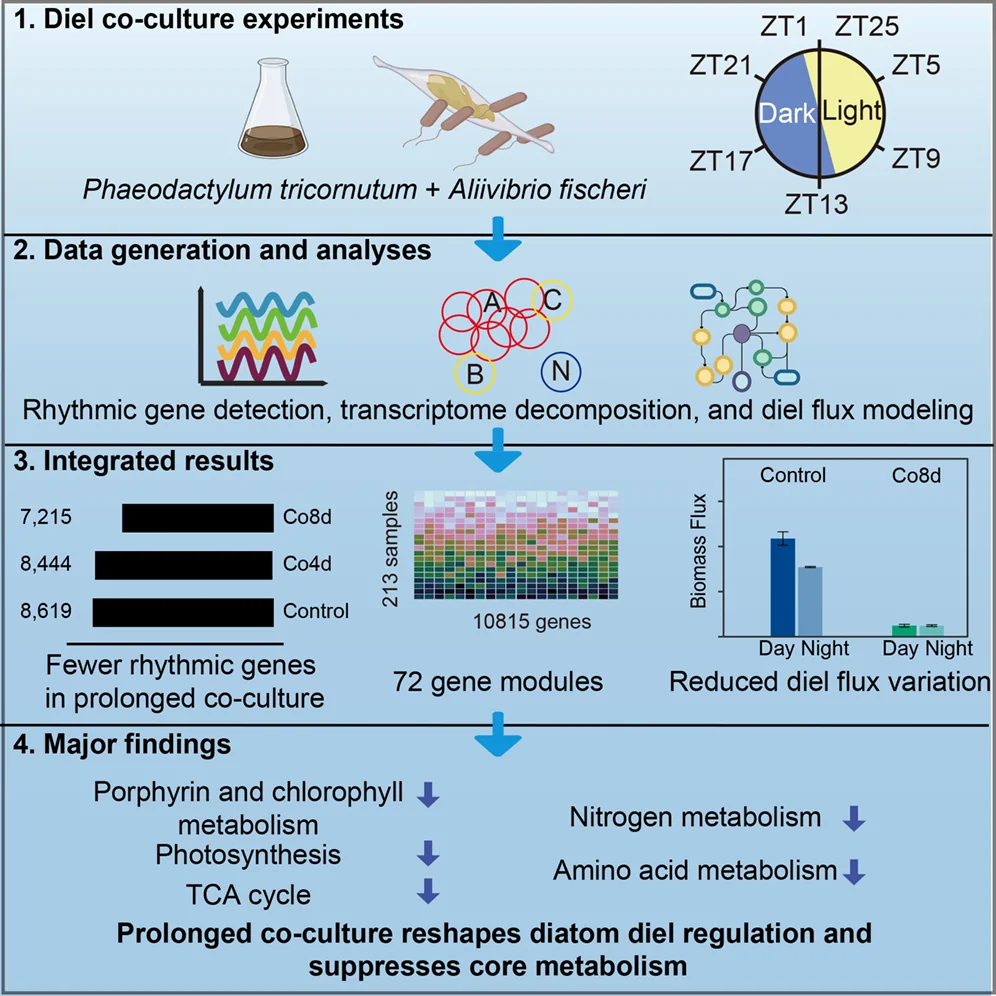

## Teaser

Microbiology; Systems biology; Transcriptomics

## Introduction

Diatoms are among the most important groups of marine microalgae. Originating approximately 250 million years ago, they now comprise an estimated 100,000 to 200,000 extant species and contribute ∼20% of annual global carbon fixation.^1^^,^^2^ Diatoms occur in diverse aquatic habitats and experience predictable daily changes in light availability.^3^^,^^4^ To anticipate these predictable oscillations, diatoms evolved endogenous circadian clocks that orchestrate their physiology and metabolism.^5^^,^^6^^,^^7^ Such temporal regulation may help coordinate physiological and metabolic processes with predictable daily changes in light and nutrient availability.^5^ Diel transcriptomic analyses of cultured diatoms have delineated pervasive circadian gene expression,^4^^,^^8^^,^^9^^,^^10^^,^^11^ revealing how circadian control of metabolism coordinates energy flux with growth. These rhythms are shaped by external cues such as light regime and nutrient status.^9^^,^^12^^,^^13^ They are also influenced by internal regulatory mechanisms, including GSH redox potential, MYB transcription factors, and photoreceptors.^14^^,^^15^^,^^16^^,^^17^

However, pure culture models fail to recapitulate the complex ecological interactions that characterize natural marine systems. A substantial body of laboratory and field studies has documented diverse diatom-bacteria interactions,^18^ including species-specific bacterial associations,^19^ bacterial effects on diatom growth and physiology,^20^^,^^21^ nutrient and metabolite exchange,^18^^,^^22^ extracellular polymeric substance production,^21^ and chemically mediated microbiome structuring.^23^ Despite this progress, how bacteria reshape diatom temporal gene-expression programs remains largely unexplored. To address this critical gap, we employed *Phaeodactylum tricornutum*, a widely used coastal and benthic-associated model diatom. As one of the first diatoms to be sequenced, *P. tricornutum* exhibits mixotrophic capacity and possesses a genome enriched with horizontally acquired bacterial genes, making it a tractable system for investigating algal-bacterial crosstalk.^1^^,^^24^^,^^25^^,^^26^ We paired this diatom with the heterotrophic bacterium *Aliivibrio fischeri* as a defined co-culture partner. The strain was initially selected in part because its bioluminescent phenotype allowed us to test whether bacterial presence might influence algal physiology under diel conditions.^27^^,^^28^ However, co-culture with *A. fischeri* inhibited rather than promoted *P. tricornutum* growth; we therefore focused our subsequent analyses on the effects of bacterial association on diel physiology and transcriptomic rhythmicity.

Elucidating how bacterial association reshapes diatom diel regulation requires analysis of rhythmic transcription, metabolic-state changes, and coordinated gene modules from time-series transcriptomes.^29^^,^^30^^,^^31^^,^^32^^,^^33^ Yet, robustly extracting independently regulated modules from high-dimensional expression datasets remains challenging for conventional ICA-based workflows, particularly when issues of component reproducibility and dimensionality selection must be resolved across repeated runs.^34^^,^^35^ To overcome this limitation, we incorporated DeepCluster ICA, a method that retains linear FastICA decomposition but improves post-ICA component aggregation through latent embedding and probabilistic clustering, therefore enabling more scalable and robust iModulon discovery.^36^^,^^37^^,^^38^

**Figure 1:**
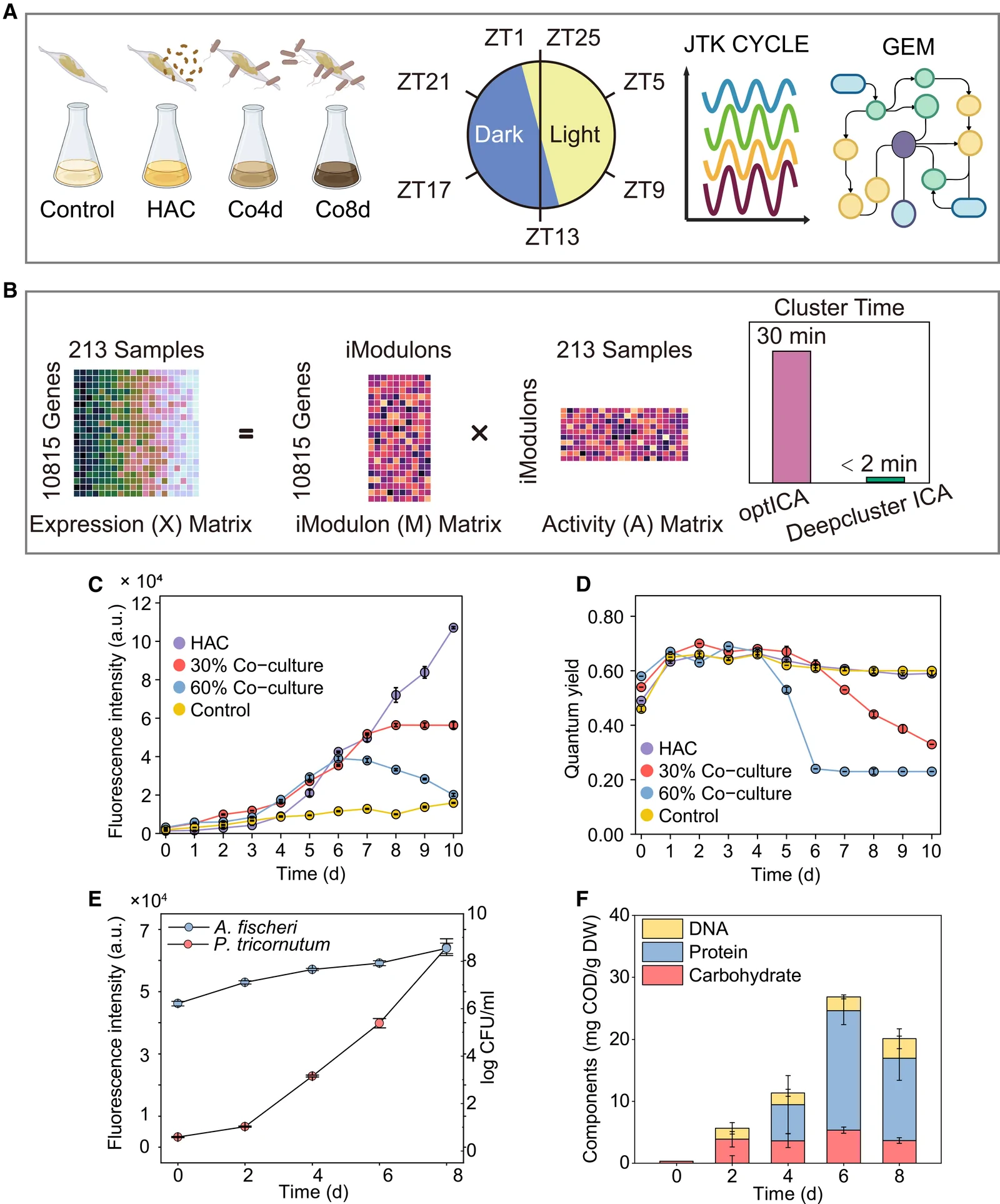
Experimental design and metabolic responses of *P. tricornutum* under bacterial co-culture stress (A) Design of four different cultivation conditions for circadian rhythms analysis and genome-scale metabolic modeling (GEM). (B) ICA-based transcriptome decomposition: expression matrix (X), iModulon gene weights (M), and temporal activation profiles (A), clustered by optICA and DeepCluster ICA. (C and D) Effects of varying marine broth concentrations on Fluorescence intensity (a.u.) (C) and quantum yield (D) of *P. tricornutum* under control, HAC, and bacterial co-culture conditions. (E) Dynamics of algal cell density (fluorescence intensity, a.u.) and bacterial abundance (*A. fischeri*, log CFU/mL) in 30% marine broth co-culture. (F) Extracellular polymeric substance (EPS) composition over time in the 30% marine broth co-culture of *P. tricornutum* with A. fischeri. Quantities are expressed in milligrams of chemical oxygen demand (COD) per gram of biomass dry weight (DW). Abbreviations: Control, axenic culture of *P. tricornutum* on EASW medium; HAC, heterotrophic/autotrophic axenic culture of *P. tricornutum* on co-culture medium; Co4d, 4-day co-culture of *P. tricornutum* with A. *fischeri* on co-culture medium; Co8d, 8-day co-culture of *P. tricornutum* with A. *fischeri* on co-culture medium. Data in (C–F) are presented as mean ± SD from three biological replicates (*n* = 3), where n represents independent cultures. Data points represent the mean of three biological replicates, and error bars indicate the standard deviation (SD).

To address this knowledge gap, we characterize the diurnal transcriptome of *P. tricornutum* under both axenic culture and algal-bacterial co-culture systems (Figures 1A and 1B). Using a defined co-culture with *A. fischeri*, we investigated how bacterial presence alters the diatom’s transcriptional rhythmicity across a light-dark cycle. We first identified rhythmic transcripts and pathway-level temporal dynamics, then integrated transcriptomic data with genome-scale metabolic modeling to infer time-resolved biomass fluxes, and finally applied ICA-based transcriptome decomposition to resolve independently regulated gene modules. Collectively, this strategy enables a systems-level analysis of diel physiological and transcriptomic responses in a defined diatom-bacterium co-culture system.

## Results

### Bacterial coexistence establishes competitive rather than synergistic dynamics in *Phaeodactylum tricornutum* cultures

This study first designed and defined an algae-bacteria co-culture system (Figures 1A and 1B). In the co-culture system, moderate supplementation with marine broth (30% v/v) significantly increased the cellular density of *P. tricornutum* (Figures 1C and 1E). In contrast, excessive supplementation (60% v/v) initially boosted algal cell density relative to the control group in one week of cultivation. However, it subsequently caused a rapid decline in cell density, with the quantum yield falling to approximately 0.2 (Figures 1C and 1D). Although *P. tricornutum* can be maintained in pure culture using tryptone and urea as nitrogen sources supplemented with glycerol as carbon substrate,^26^ dark-phase experiments revealed non-utilization of yeast extract and tryptone by the diatom (Figure S1A), though mixotrophic metabolism resumed under illumination (Figure S1B).

To test whether the growth enhancement caused by 30% marine broth supplementation was due to bacterial effects or the medium itself, we established a heterotrophic/autotrophic axenic culture (HAC) group, in which *P. tricornutum* was grown alone in EASW with 30% marine broth under the same 12 h:12 h light-dark cycle and without *A. fischeri*. In the axenic HAC condition, *P. tricornutum* sustained high growth rate through day 10, consistent with its known mixotrophic capacity,^39^ with QY remaining between 0.6 and 0.7 during this period (Figure 1D). The day-8 axenic control also retained a high QY (0.60 ± 0.01). In contrast, Co8d showed a marked decline in QY, indicating that the late-stage deterioration was not simply a consequence of culture age (Figure 1D). These results are more consistent with a competitive effect in co-culture than with a growth-promoting role of *A. fischeri*. Photosynthetic pigments were measured as auxiliary indicators of algal culture status. Chl *a*, Chl *c*, and carotenoid concentrations per culture volume generally followed the 488/695 nm chlorophyll fluorescence-based growth trends (Figures S2A–S2C). Nutrient analysis revealed elevated initial nitrogen (N) and phosphorus (P) levels in co-culture systems compared to controls, attributable to supplemented peptone and yeast extract. Nitrogen content showed rapid depletion during the first 4 days, followed by a gradual decrease, while phosphorus was completely consumed within the first day (Figures S3A and S3B). This accelerated P depletion likely resulted from preferential uptake and storage by the high initial algal biomass.

Contrary to our initial hypothesis that bioluminescence emission from *A. fischeri* might stimulate dark-phase growth of *P. tricornutum*, no such positive effects were observed (Figure S1). The experimental outcomes collectively suggest that the growth-promoting effects of moderate marine broth supplementation primarily derive from nutritional components rather than direct bacterial positive interactions. The observed nutrient depletion patterns and pigment dynamics suggest early-phase competition for amino acid resources between *P. tricornutum* and *A. fischeri*, establishing fundamental constraints in algal-bacterial consortia development.

### Bacterial surface colonization and prolonged co-culture drive EPS depletion and severe photosynthetic dysregulation

Scanning electron microscopy (SEM) analysis demonstrated significant colonization of *P. tricornutum* cell surfaces by *A. fischeri*, with associated filamentous structures likely corresponding to extracellular polysaccharides (EPS) produced by either organism (Figure S4). Quantitative assessment of EPS production in the 30% marine broth co-culture of *P. tricornutum* with *A. fischeri* revealed a peak at day 6, followed by a marked reduction by day 8 (Figure 1F). This temporal pattern may stem from multiple factors: nutrient depletion diminishing EPS biosynthesis efficiency, biomass accumulation diluting relative EPS concentrations, and potential bacterial catabolism of EPS as an auxiliary carbon source under nutrient-limiting conditions.

**Figure 2:**
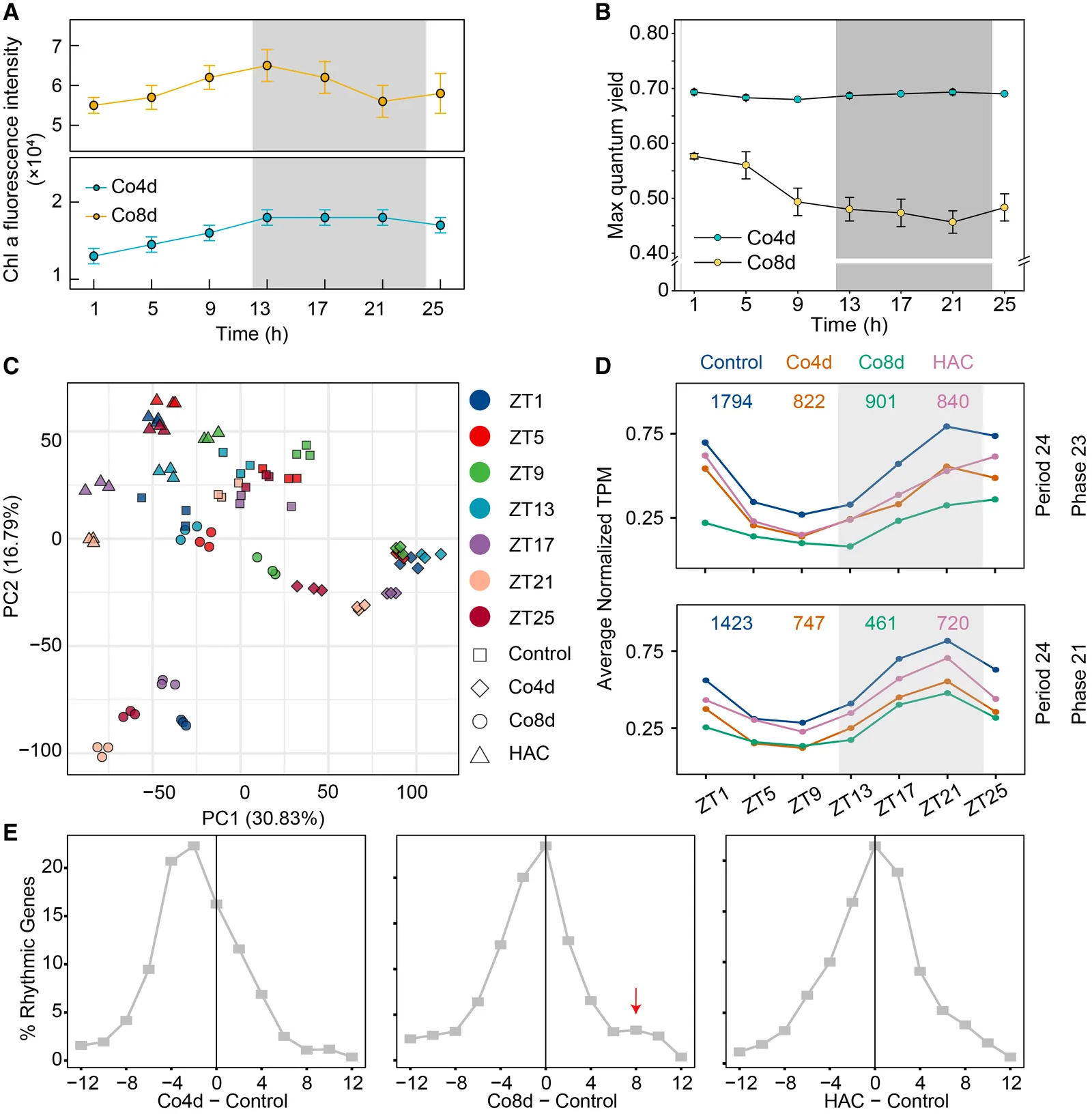
Time-series physiological and transcriptomic analysis of *P. tricornutum* under bacterial co-culture stress (A) Diurnal dynamics of chlorophyll *a* (Chl a) fluorescence intensity and (B) maximum quantum yield (QY) of photosystem II. Data points represent the mean of three biological replicates, and error bars indicate the standard deviation (SD). (C) Principal component analysis (PCA) of transcriptomes sampled across the four experimental groups at different time points (ZT1 to ZT25). (D) Representative rhythmic expression patterns of JTK-identified genes. The plots show two representative groups with a 24-h period and peak phases at ZT21 or ZT23. Each line represents the average normalized TPM (Transcripts Per Million) for all genes in that group. The numbers listed above the plots indicate the total count of rhythmic genes identified in each condition. Comprehensive temporal expression profiles for all rhythmic genes are provided in Figures S5 and S6. (E) Distribution of phase differences of shared rhythmic genes in the Co4d, Co8d, and HAC groups relative to the control group. Negative values indicate phase advance and positive values indicate phase delay. The red arrow indicates a minor +8 h shoulder in Co8d. The shaded regions indicate the dark period (11 h–23 h). Control, axenic culture of *P. tricornutum* on EASW medium; HAC, heterotrophic/autotrophic axenic culture of *P. tricornutum* on co-culture medium; Co4d, 4-day co-culture of *P. tricornutum* with *A. fischeri* on co-culture medium; Co8d, 8-day co-culture of *P. tricornutum* with *A. fischeri* on co-culture medium. Data in (A and B) are presented as mean ± SD from three biological replicates (*n* = 3), where n represents independent cultures.

To elucidate diurnal physiological adaptations of *P. tricornutum* in algae-bacteria batch co-culture systems, we performed continuous 24-h monitoring at two critical time points (Co4d and Co8d). Co4d analyses revealed robust photosynthetic activity, characterized by progressive daytime elevation of chlorophyll *a* fluorescence followed by nocturnal attenuation, consistent with previous reports.^40^ Concurrently, quantum yield (QY) remained stable across the diel cycle in Co4d, with values consistently close to 0.7 (Figure 2B). In stark contrast, Co8d profiles displayed progressive QY deterioration from an initial 0.57, culminating in pre-dawn minima with only partial recovery following 1-h light exposure (Figures 2A and 2B). These divergent patterns indicate substantial impairment of photosynthetic functionality in *P. tricornutum* during advanced co-cultivation stages. Consistent with the persistently high QY observed in the HAC condition and the day-8 axenic control, the pronounced decline in Co8d (Figure 1D) suggests that this late-stage deterioration was associated with prolonged co-culture rather than cultivation time alone.

### Co-culture-associated changes in phase distribution and transcriptional rhythmicity

Principal component analysis (PCA) of the transcriptomic profiles across four experimental groups (Control, HAC, Co4d, Co8d) with 84 samples revealed distinct clustering patterns (Figure 2C). Notably, the Control and HAC groups exhibited closer proximity, while the Co8d group was the most distant from the other groups. Interestingly, within the Co8d group, samples collected at ZT5, ZT9, and ZT13 (corresponding to midday and dusk conditions) were closer to the other groups compared to samples from ZT1, ZT17, ZT21, and ZT25. This pattern indicates diel variation in the degree of transcriptomic separation within Co8d.

Using the JTK algorithm, we identified 8,619, 9,387, 8,444, and 7,215 rhythmic genes in the Control, HAC, Co4d, and Co8d groups, respectively, representing 80%, 87%, 78%, and 67% of the transcriptome in each group (Figure S7). In line with other microalgae, including cyanobacteria and green algae, diel transcriptomic analyses of cultured diatoms have delineated pervasive circadian gene expression.^8^^,^^41^^,^^42^^,^^43^^,^^44^^,^^45^^,^^46^^,^^47^^,^^48^^,^^49^ The proportion of rhythmic genes was highest in the HAC group. UpSet analysis further revealed that 4,621 rhythmic genes were shared among all four groups, followed by 1,778 genes shared among all groups except the Co8d group, and 866 genes shared among all groups except the Co4d group (Figure S7). These findings indicate a decline in the proportion of rhythmic genes during algal-bacterial co-cultivation, with prolonged co-cultivation leading to a more pronounced reduction.

The majority of rhythmically expressed genes displayed robust 24-h periodicity, with peak transcriptional activity clustered at ZT21 or ZT23. The relative abundance of these rhythmically expressed genes varied across experimental groups, constituting 37% in the Control group, 17% in the HAC group, 19% in the Co4d group, and 19% in the Co8d group (Figures 2D and S6). Notably, genes exhibiting 12-h oscillatory patterns demonstrated marked quantitative differences, with the Co8d group showing a reduced number of such rhythmically expressed entities compared to both the other three experimental groups and the 24-h oscillatory profiles (Figures S5 and S6).

Analysis of circadian phase shifts revealed distinct patterns among the three conditions (Figure 2E). In the early stage of co-cultivation (Co4d), a substantial subset of rhythmic genes showed a phase advance of approximately −4 h (Figure 2E). In contrast, the Co8d group displayed a delayed distribution, with peak shifts concentrated around +8 h (Figure 2E). By comparison, the HAC group showed a narrow and nearly symmetrical distribution centered around zero phase difference (Figure 2E). These results indicate that the timing of rhythmic gene expression differed markedly between the early and late co-culture states, whereas the axenic medium control showed relatively stable phase alignment.

The Tau index, which quantifies biological rhythm regularity, showed that rhythmic genes consistently exhibited higher Tau indices than non-rhythmic genes across all groups (Wilcoxon test, *p* < 0.05), reinforcing their circadian nature (Figure S8). Interestingly, the Tau indices were higher in the HAC, Co4d, and Co8d groups compared to the Control (*p* < 0.05), indicating amplified diel metabolic disparities under these conditions. Furthermore, the Co4d and Co8d groups exhibited higher Tau indices than the HAC group (*p* < 0.05), indicating more pronounced circadian disruption under co-culture conditions.

### KEGG profiling reveals time-dependent suppression of core metabolism in *P. tricornutum* under bacterial coexistence

**Figure 3:**
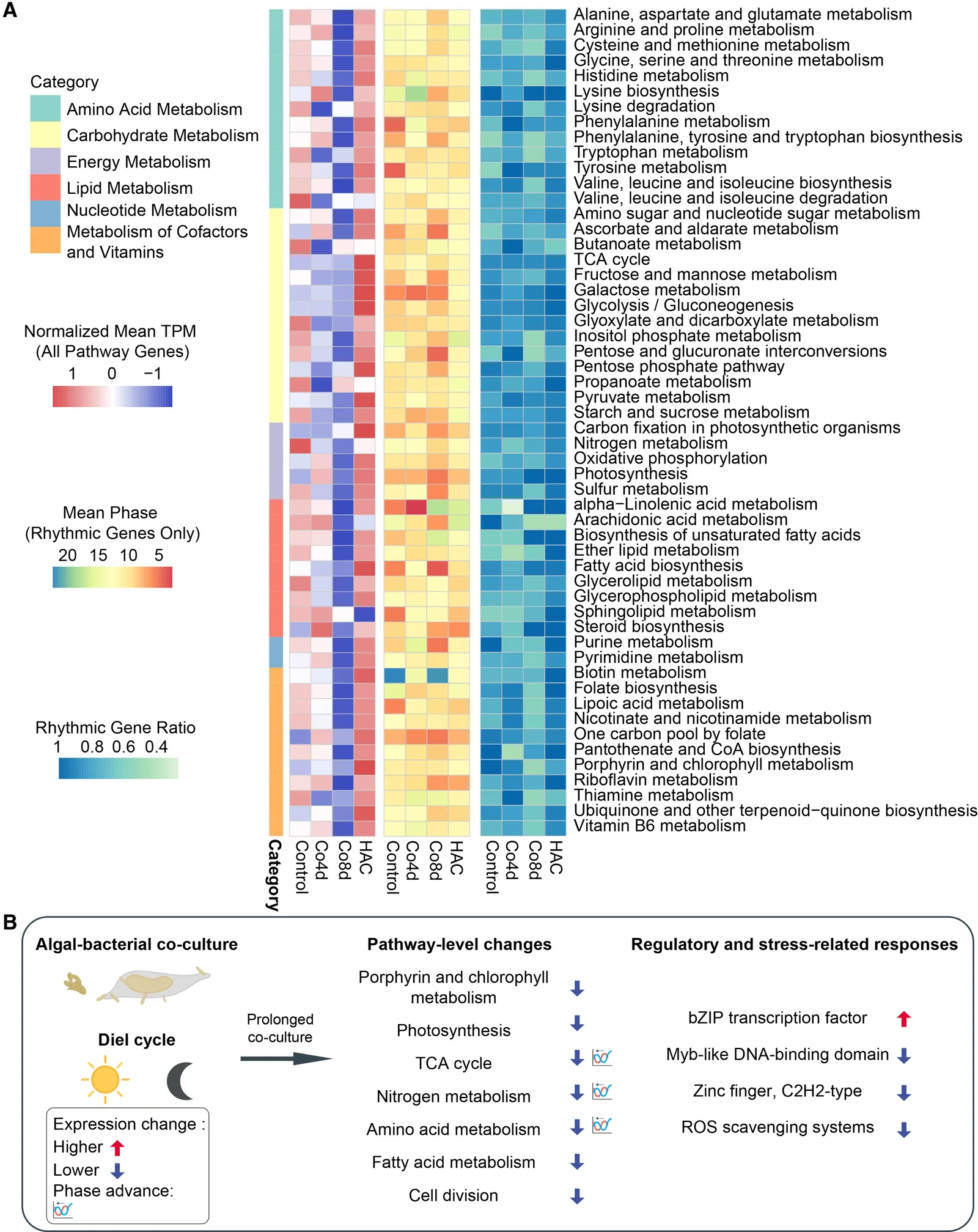
Diel regulation of metabolic pathways in *P. tricornutum* under bacterial co-culture stress (A) A comprehensive heatmap illustrates the expression profiles, rhythmic properties, and expression phases of major metabolic pathways, organized by functional category (left color bar). The heatmap displays, for each experimental condition (control, Co4d, Co8d, HAC), the pathway-level mean expression calculated from TPM values averaged across biological replicates at each condition and time point and then min-max normalized across all samples (left image; red, high; blue, low), the mean phase (time of peak expression in Zeitgeber hours, ZT) for rhythmic genes (middle image), and the proportion of rhythmic genes within the pathway (right image; darker blue indicates a higher ratio). (B) Schematic summary of pathway-level, regulatory, and stress-related responses of *P. tricornutum* under prolonged co-culture. Red upward arrows indicate higher expression, blue downward arrows indicate lower expression, and the phase icon indicates phase advance. Control, axenic culture of *P. tricornutum* on EASW medium; HAC, heterotrophic/autotrophic axenic culture of *P. tricornutum* on co-culture medium; Co4d, 4-day co-culture of *P. tricornutum* with *A. fischeri* on co-culture medium; Co8d, 8-day co-culture of *P. tricornutum* with *A. fischeri* on co-culture medium.

Recognizing that conventional pairwise enrichment analysis is often insufficient for comprehensively revealing circadian-regulated gene patterns within complex transcriptomic data, we employed a more dynamic rhythmic analysis. This approach was applied to a curated set of 54 key KEGG pathways that represent critical biological processes. These pathways were further organized into six major metabolic categories: amino acid metabolism, carbohydrate metabolism, energy metabolism, lipid metabolism, nucleotide metabolism, and metabolism of cofactors and vitamins, providing a holistic view of the cell’s rhythmic metabolic landscape (Figures 3A and S9–S12).

The rhythmic analysis revealed distinct metabolic responses to the bacterial co-culture over time (Figure 3A). A striking pattern emerged in the Co8d group, where the majority of metabolic pathways exhibited significant downregulation in mean expression, indicative of severe physiological stress in the late co-culture condition by day 8. In contrast, gene expression in the Co4d condition remained largely comparable to that in the Control, yet was suppressed relative to the heterotrophic/autotrophic axenic culture (HAC), suggesting that co-culture-associated effects, including possible nutrient competition, had already emerged. Beyond expression levels, the co-culture was associated with a substantial temporal reorganization of pathway-level rhythmic features. In the Co8d group, the mean phase of rhythmic genes displayed a notable delay, shifting from the dark phase toward the light phase, a phenomenon particularly pronounced in amino acid and carbohydrate metabolism pathways (Figures 3A and S9–S11). Consistent with a general disruption of cellular rhythmicity, the proportion of rhythmic genes within these pathways was lowest in the Co8d group and highest in the HAC group, mirroring the overall scale of the rhythmic transcriptomes observed previously.

The Co8d culture displayed the lowest average expression, confirming a state of suppressed metabolism (Figure 3A). Critically, a significant phase advancement of peak expression was observed, shifting from early night (∼ZT13) to late day (∼ZT9) in the alanine/aspartate/glutamate, nitrogen, and TCA cycle (tricarboxylic acid cycle) pathways (Figures 3A and S9). This temporal realignment is consistent with a stress-associated metabolic adjustment, in which daytime resource allocation may become relatively prioritized, potentially at the expense of overall metabolic efficiency. The photosynthesis pathway, while also suppressed in Co8d, maintained its peak expression during the light phase as expected (Figures 3A and S11A). Finally, in the nitrogen metabolism pathway, expression was suppressed not only in co-cultures but also in the HAC condition compared to the Control (Figures 3A and S11A). These results suggest that the amino acids present in the enriched co-culture and HAC media reduced the alga’s reliance on assimilating inorganic nitrogen, a factor related to nutrient availability rather than direct bacterial interaction alone. While major transcription factor families exhibited strongly reduced and flattened expression in Co8d cultures (Figure S13), bZIP transcription factors were notably upregulated, particularly from the late day phase (ZT9) onwards (Figure S13C). Beyond their roles in stress adaptation, recent studies in *P. tricornutum* have revealed that subsets of bZIP transcription factors display pronounced rhythmic expression patterns and contribute to diurnal regulation of key metabolic processes, including laminarin and TCA cycle metabolism.^50^^,^^51^ Specifically, bZIP14 has been identified as a regulator orchestrating metabolic shifts during the daily cycle and under nutrient stress,^51^ highlighting that bZIPs play dual roles in both circadian metabolic regulation and biotic stress response in diatoms. Interestingly, metabolic pathways responded discretely. Core pathways, including glycolysis/gluconeogenesis, the TCA cycle, pyruvate metabolism, and amino acid degradation, maintained stable rhythmicity under bacterial co-cultivation, reflecting robust circadian control. In contrast, pathways such as arachidonic acid and inositol phosphate metabolism, thiamine, vitamin B6, pantothenate, and folate biosynthesis, as well as tryptophan, lysine, and branched-chain amino acid biosynthesis, showed decreased rhythmicity in Co8d, indicating greater sensitivity under prolonged co-culture conditions (Figure 3A).

Collectively, these findings indicate that prolonged co-culture is associated with substantial remodeling of the metabolic landscape of *P. tricornutum*, with progressive dysregulation from Co4d to Co8d (Figure 3B). The core of this reprogramming is a critical breakdown of metabolic temporal partitioning. Under severe stress in the Co8d condition, the diatom is forced into an energetically inefficient state of ‘metabolic convergence’, running key biosynthetic and catabolic processes such as the TCA cycle concurrently with photosynthesis during the light phase. While this strategy likely serves to fuel immediate defensive responses and repair cellular damage, it sacrifices long-term metabolic efficiency, as evidenced by the dampened amplitude and overall suppression of these pathways. Ultimately, this loss of temporal organization is consistent with a shift from relatively growth-oriented metabolism toward a more stress-associated state, which aligns with the observed decline in algal vitality under prolonged co-culture conditions.

### Genome-scale metabolic modeling suggests a biphasic shift and diel redistribution of predicted carbon flux under co-culture conditions

**Figure 4:**
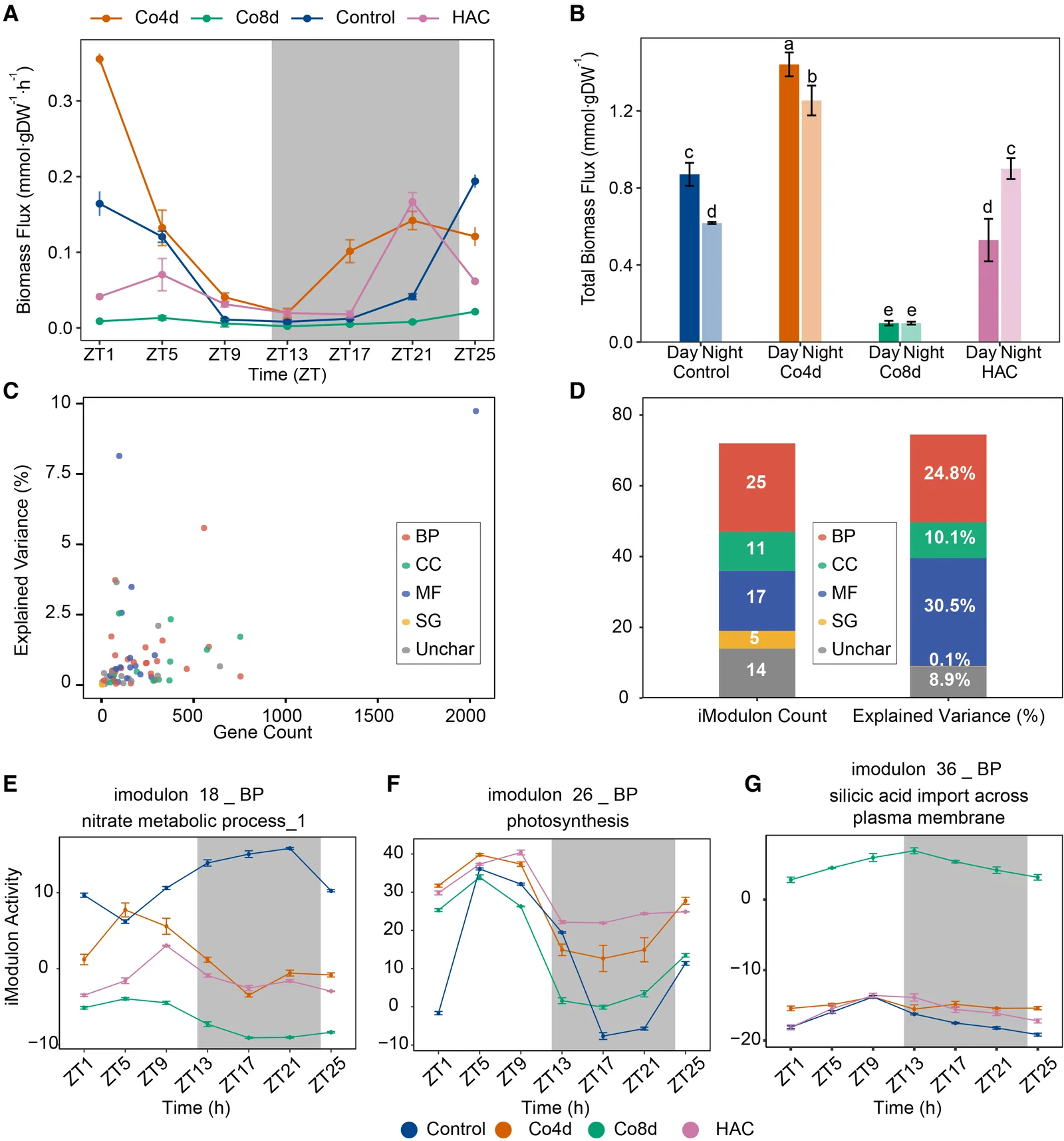
Integration of GEM and ICA identifies rhythmic metabolic and regulatory modules in algae-bacteria co-cultures (A) Biomass flux profiles across the diurnal cycle modeled by GEM with transcriptomic constraints. Gray shading denotes nighttime. (B) Daytime and nighttime total biomass flux of each group over 24 h. Different letters denote significant differences (*p* < 0.05). (C) Distribution of iModulons by gene count and explained variance, colored by 5 iModulon categories. (D) iModulon count and explained variance by category. (E–G) Time-resolved expression patterns of three functionally distinct iModulons: (E) Nitrogen metabolism-associated module, (F) Photosynthesis-related module, (G) Silicic acid transmembrane transport module, highlighting differential activation across experimental groups (Control, HAC, Co4d, Co8d). Abbreviations: BP, biological process iModulon; MF, molecular function iModulon; CC, cellular component iModulon; SG, single-gene iModulon; Unchart, iModulon with uncharacterized function. Control, axenic culture of *P. tricornutum* on EASW medium; HAC, heterotrophic/autotrophic axenic culture of *P. tricornutum* on co-culture medium; Co4d, 4-day co-culture of *P. tricornutum* with *A. fischeri* on co-culture medium; Co8d, 8-day co-culture of *P. tricornutum* with *A. fischeri* on co-culture medium. Data in (A), (B), and (E)–(G) are presented as mean ± SD from three biological replicates (*n* = 3), where n represents independent cultures. Data points represent the mean of three biological replicates, and error bars indicate the standard deviation (SD).

By integrating transcriptomic data with genome-scale metabolic modeling, we systematically analyzed dynamic changes in biomass flux and gene expression in *P. tricornutum* under four different culture conditions across the diurnal cycle (Figures 4A and 4B). The Co4d, HAC, and Control groups exhibited clear diel rhythms, with fluxes lowest around dusk (ZT13) and highest near dawn (ZT1 and ZT25), reflecting pronounced responses to light-dark transitions. The HAC group exhibited higher biomass flux at night than during the day, revealing a capacity for light-independent metabolism driven by the assimilation of supplemental amino acids and peptone. In contrast, the Co8d group showed minimal variation in biomass flux, indicating a loss of rhythmic metabolic activity.

Total biomass flux analysis (Figure 4B) showed that Co4d had the highest 24-h productivity, followed by the lower fluxes in the Control and HAC groups, with Co8d showing the lowest flux overall. The relatively high flux in Co4d may reflect the nutrient-enriched conditions and sustained metabolic activity present during the early stage of co-culture. In contrast, the sharp decline in Co8d points to a pronounced reduction in metabolic performance during prolonged co-culture, likely associated with progressive nutrient depletion and other co-culture-related effects, including bacterial interaction. EPS components increased most rapidly between days 4 and 6 and then declined from days 6–8, a pattern that broadly matched the modeled biomass flux changes (Figure 1F).

Co-culture with *A. fischeri* was associated with a pronounced biphasic shift in the predicted carbon fixation dynamics of the diatom. After 4 days of co-culture (Co4d), the total 24-h biomass flux increased by approximately 81% relative to the axenic control, with daytime and nighttime fluxes rising by 66% and 103%, respectively. By contrast, prolonged co-culture (Co8d) led to a marked decline, reducing fluxes by 87% (24-h), 89% (daytime), and 84% (nighttime). When averaged over the entire co-culture period, net flux decreased modestly by 2.91% over 24 h, reflecting a distinct diel redistribution: daytime flux dropped by 11.59%, while nighttime flux increased by 9.31%. This shift was driven largely by the strong Co4d response, in which fluxes related to photosynthesis and fatty acid biosynthesis were significantly elevated at night (Figures 3A, S11A, and S11B). Together, these patterns are consistent with increased dark-phase allocation of photosynthetically derived carbon to storage and structural lipids during early co-culture.

Taken together, these flux patterns suggest that prolonged co-culture alters not only the overall level of predicted biomass production, but also the timing of carbon allocation across the diel cycle. In this sense, the late-stage Co8d group appears to reflect a redistribution of metabolic priorities under co-culture-associated stress, rather than a simple uniform decline in activity. Although the present system is a defined laboratory co-culture, these results raise the possibility that microbial interactions may shape daily patterns of algal carbon use in temporally structured environments. Determining whether similar diel biases occur in natural phytoplankton-bacteria assemblages will require further study in more ecologically representative systems.

### ICA decomposed the *P. tricornutum* transcriptome into 72 iModulons

We compiled a comprehensive dataset of 213 RNA-seq expression profiles for *P. tricornutum*, including 84 newly generated samples from this study and 129 published datasets from our previous study (Figure 1B). Following standardized processing protocols, we constructed a gene-by-sample expression matrix comprising 10,815 genes and 213 samples. Using independent component analysis, we systematically identified 72 distinct iModulons encompassing 959 genes (Figure 4C). These transcriptional modules were functionally categorized into five major classes: regulatory, genomic, functional, single-gene, and uncharacterized. To quantify the biological significance of each iModulon, we calculated the explained variance, a metric reflecting its contribution to transcriptional variability within the dataset. Collectively, the 72 iModulons accounted for 74.5% of the total expression variation, demonstrating their comprehensive capacity to capture global transcriptional dynamics.

Functional annotation identified 53 iModulons with significant Gene Ontology (GO) enrichment, comprising 17 molecular function (MF), 25 biological process (BP), and 11 cellular component (CC) terms. In addition, 14 iModulons lacked functional characterization (Unchar), and 5 were classified as single-gene (SG) modules (Figure 4C). MF and BP iModulons accounted for the majority of explained transcriptomic variance, with MF modules containing larger gene sets and contributing most significantly (Figures 4C and 4D). The proportion of rhythmic genes among the five major iModulon categories was lowest in the Co8d group and highest in the HAC group (Figure S14A). Modulon activity also exhibited rhythmicity, evident in BP, CC, and MF categories, while uncharacterized iModulons (Unchar) displayed notably lower rhythmicity (Figure S14B).

Temporal profiling revealed rhythmically activated iModulons displaying phase-specific expression dynamics comparable to KEGG pathway oscillations. Notably, iModulon_18_BP (“nitrate metabolic process”) exhibited elevated expression in control groups relative to co-culture conditions, with algal-bacterial cocultivation inducing a phase shift in its rhythmic activity, advancing the cycle peak from nighttime to daytime (ZT5), preceding the ZT9 peak observed in HAC groups (Figure 4E). Concurrently, iModulon_36_BP (“silicic acid transmembrane transport”) displayed Co8d-specific transcriptional amplification, indicative of stress-activated silicon shell biosynthesis, a metabolic adaptation undetected by conventional KEGG pathway analysis (Figure 4F).

Among the five single-gene iModulons, only SG_1 (Phatr3_J37403), annotated as encoding a senescence-associated protein (Table S2), exhibited differential regulation under stress conditions. Quantitative analysis revealed significantly elevated expression of SG_1 in the Co8d group compared to other experimental cohorts, with notable expression fluctuations observed in Co4d samples (Figure S15A). This pattern is consistent with a late-stage co-culture-associated stress response accompanied by altered senescence-associated regulation, potentially indicating a survival strategy through modified senescence regulation under prolonged environmental challenge. These findings collectively demonstrate the superior capability of independent component analysis (ICA) in revealing novel stress-responsive regulatory mechanisms that transcend the limitations of standard pathway annotation frameworks.

### A refined DeepCluster ICA algorithm delivers enhanced performance

**Figure 5:**
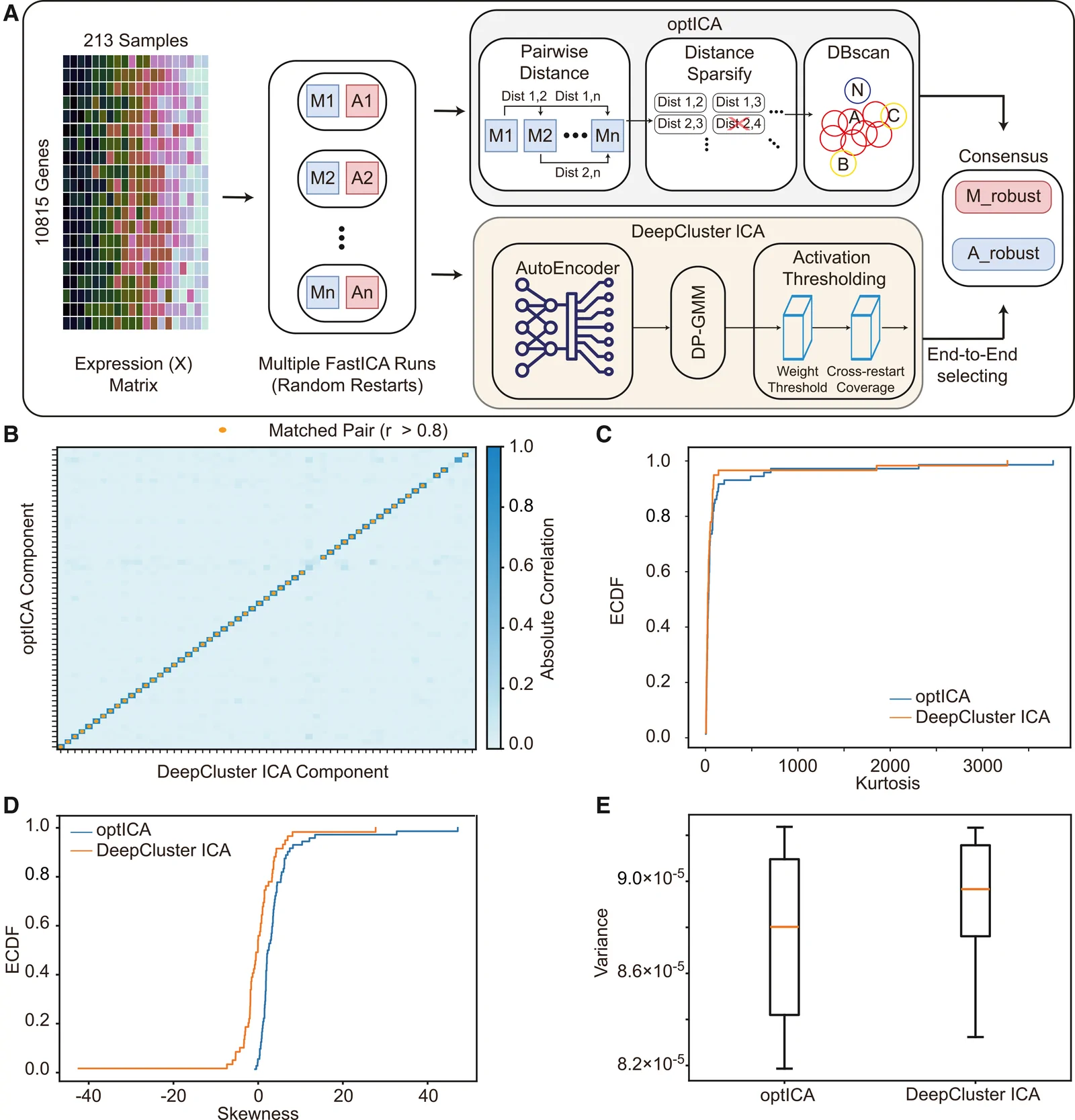
Performance comparison between DeepCluster ICA and optICA for identifying robust independent components (A) A schematic diagram illustrates the workflows for both methods. Both start with multiple FastICA runs with random restarts on a gene expression matrix (10,815 genes × 213 samples). The optICA method identifies robust components by clustering results from multiple runs based on pairwise distance calculations and DBSCAN. In contrast, DeepCluster ICA utilizes an end-to-end deep learning framework that integrates an autoencoder, a Dirichlet Process Gaussian Mixture Model (DP-GMM), and activation thresholding to select stable components. (B) Heatmap shows the absolute Pearson’s correlation between components derived from DeepCluster ICA (x axis) and optICA (y axis). Orange dots on the diagonal denote matched pairs with a correlation coefficient (*r*) > 0.8. (C) Empirical cumulative distribution function (ECDF) of kurtosis values for the sets of components identified by each method. (D) ECDF of skewness values for the components identified by each method. (E) Boxplot comparing the distribution of variance for components derived from optICA and DeepCluster ICA. The central line represents the median, the box bounds define the interquartile range (IQR), and the whiskers extend to 1.5 times the IQR. Here, *n* represents the number of components identified by each method (optICA, *n* = 59; DeepCluster ICA, *n* = 68).

To compare independent component analysis methods for transcriptomic decomposition, we first implemented both optICA and DeepCluster ICA on the same gene expression matrix consisting of 10,815 genes across 213 samples. optICA relies on multiple FastICA runs with random initializations, followed by pairwise distance computation and DBSCAN-based clustering to identify robust components via consensus across runs (Figure 5A). In contrast, DeepCluster ICA replaces this pipeline with a deep neural network that integrates an autoencoder for nonlinear feature extraction, a Dirichlet Process Gaussian Mixture Model (DP-GMM) for adaptive component modeling, and activation-based thresholding to select non-Gaussian signals (Figure 5A).

Comprehensive numerical and visual analyses indicate that DeepCluster ICA is better suited than optICA when the cross-restart structure is predominantly nonlinear (Figure S16). Across multiple restarts, PCA revealed no linear separation for either matrix (Figures S16A and S16B), whereas UMAP and t-SNE of the A matrix (sample-activity profiles) exhibited clear clustering (Figures S16C and S16E), confirming the manifold structure that linear projections fail to capture. By contrast, two-dimensional projections of the M matrix, the gene-loading matrix with per-gene weights for each component, appeared dispersed with only weak nonlinear separation (Figures S16D and S16F), a common outcome under high dimensionality, sparsity, and feature dilution without global alignment and feature selection; under these conditions, low-dimensional views tend to under-expose cross-restart concordance of gene loadings.

In response to this geometry, DeepCluster ICA models and clusters on the M matrix by first learning a nonlinear autoencoder embedding that preserves local neighborhoods and denoises high-dimensional loadings, thereby making cross-restart structure more linearly separable in the latent space. Clustering is then performed with a Dirichlet-process Gaussian mixture, which supplies soft assignments, mixture weights, and automatic complexity control, features that handle heterogeneous densities and overlapping clusters more reliably than the single-scale density thresholding used by DBSCAN in optICA. Combined with mixture-weight filtering and a cross-restart coverage filter that retains only clusters observed in a required fraction of ICA restarts, the procedure down-weights or excludes low-confidence components and produces more reproducible consensus features across restarts.

The empirical cumulative distribution functions (ECDFs) of kurtosis are nearly superimposed, with optICA exhibiting a slight, yet discernible, extreme tail (Figure 5C). These results indicate that a few optICA components retain exceptionally heavy-tailed distributions, which may represent outlier signals. This observation is further supported by the skewness ECDF (Figure 5D): optICA retains a small subset of highly asymmetric signals, whereas these extremes are almost absent in DeepCluster ICA. DeepCluster ICA achieves a smoother skewness distribution, suggesting its ability to filter rare, highly skewed signals without compromising the bulk of biologically relevant variation. Crucially, the variance distributions, as shown in the boxplots (Figure 5E), are statistically indistinguishable. These results demonstrate that the deep-learning workflow achieves an order-of-magnitude speed improvement without introducing additional component noise. In this study, DeepCluster ICA maintains the optICA-derived non-Gaussian structure and reduces the influence of extreme or sparsely represented components, achieving a small improvement in statistical independence while maintaining biological interpretability at the pathway level. Furthermore, assessing internal decorrelation, DeepCluster ICA achieves a slightly lower average absolute internal correlation (0.0034) compared to optICA (0.0038) (Figure 5B). This difference indicates a marginally superior capacity to minimize linear dependencies among the extracted components. Such improved decorrelation translates to reduced redundancy and a higher degree of statistical independence, which is particularly advantageous for downstream applications such as unsupervised clustering, visualization, and biomarker discovery. Notably, optICA explains a higher proportion of the total variance (74.5%) compared to DeepCluster ICA (69.0%). However, the primary objective of ICA is to separate statistically independent and non-Gaussian signals, rather than to maximize explained variance as in PCA. Consequently, the lower explained variance observed with DeepCluster ICA does not imply poorer performance; rather, it may indicate the presence of less unstructured noise in the results, owing to its nonlinear filtering capability. Further validation is required to assess the biological relevance of the extracted components. Moreover, the average matched absolute correlation between corresponding components from DeepCluster ICA and optICA is 0.8927 (Figure 5B), demonstrating strong agreement in the overall subspace structure captured by both methods. Specifically, the observed discrepancies are primarily restricted to a small subset of low-variance or tail components, suggesting that both approaches converge on similar dominant patterns in the data while differing in the modeling of more subtle features.

Importantly, DeepCluster ICA uses a linear ICA decomposition, with a deep autoencoder providing a nonlinear embedding of component loadings for clustering and selection. This capacity for nonlinear feature learning enhances its robustness to noise and complex variation. Furthermore, DeepCluster ICA combines FastICA-derived components with autoencoder embedding and Dirichlet-process Gaussian mixture clustering, balancing component non-Gaussianity and across-run stability through a predominantly data-driven selection procedure. In summary, DeepCluster ICA is highly scalable as it supports mini-batch optimization on GPUs, enabling efficient training on large datasets. For component selection, we replace pairwise-similarity-based clustering with an autoencoder embedding followed by Dirichlet-process Gaussian mixture clustering, which avoids constructing dense similarity matrices and reduces both runtime and memory usage while retaining the underlying linear ICA decomposition (optICA/FastICA). Consequently, this approach not only improves component independence but also significantly reduces computational costs, facilitating robust and scalable analysis for complex transcriptomic datasets.

## Discussion

In this study, *P. tricornutum* showed a progressive shift in both physiological performance and diel transcriptomic organization during co-culture with *A. fischeri*. Early co-culture was associated with phase advances in subsets of rhythmic genes, whereas the late stage showed phase delay and a marked reduction in rhythmic gene number. Similar large-scale diel transcriptional regulation has been reported in diatoms and other microalgae, and stress conditions can weaken or redistribute this temporal organization.^9^^,^^11^^,^^13^^,^^52^ In this context, the Co8d group is consistent with substantial disruption of diel regulation under prolonged co-culture, in parallel with the decline in QY and reduced post-dark recovery.

The KEGG results further suggest that prolonged co-culture was associated with weaker temporal partitioning between day- and night-biased metabolism. In *P. tricornutum* and other microalgae, light-dark cycles normally coordinate carbon fixation, storage, and utilization across the day,^11^^,^^12^^,^^31^ and diel structuring of metabolite production and microbial uptake has also been demonstrated in model microbial systems.^53^ Against this background, the altered timing of amino acid metabolism, nitrogen metabolism, and the TCA cycle in Co8d is more consistent with a stress-associated redistribution of metabolic activity than with efficient temporal separation of cellular functions. Similar weakening of diel coordination has also been reported under iron limitation.^9^^,^^13^

Genome-scale metabolic modeling likewise suggested differences in diel metabolism across the four culture conditions. Such approaches are useful for linking gene-expression states with predicted metabolic phenotypes, but the results remain model-based inferences rather than direct measurements.^30^^,^^31^^,^^32^^,^^54^ In the present study, these predictions were derived from transcriptome-constrained metabolic modeling within a simplified laboratory system and therefore should be interpreted as relative estimates rather than direct representations of intracellular carbon partitioning. In our case, the model predicted relatively higher nighttime biomass flux in HAC and reduced diel variation in Co8d, consistent with altered temporal organization of biomass-related metabolism during prolonged co-culture. These predictions should therefore be interpreted cautiously, especially because intracellular fluxes and partner-to-partner metabolite exchange were not measured directly. Accordingly, we view these results as relative predictions within a simplified laboratory system rather than as direct evidence for natural marine carbon cycling or the biological carbon pump. Future studies should test whether similar diel metabolic shifts occur in more complex phytoplankton-bacteria systems, where microbial interactions are known to influence the fate of algal carbon.^55^^,^^56^

A key limitation of this study is the absence of a matched day-8 axenic transcriptomic control, which prevents strict attribution of the late-stage transcriptional differences specifically to bacterial interaction. However, the high QY maintained in the day-8 HAC condition argues against prolonged cultivation alone as a sufficient explanation for the Co8d group. We therefore interpret the late-stage Co8d state as arising from co-culture-associated effects, likely including bacterial interaction, nutrient depletion, and changes in the culture environment, while acknowledging that growth phase may also contribute.

Conventional pathway analysis provides an overview of metabolic disruption, whereas ICA-based transcriptome decomposition can resolve independently regulated modules that are not always apparent at the pathway level.^33^^,^^57^^,^^58^ In this study, we identified 72 iModulons that captured a large fraction of transcriptomic variation, supporting the value of module-level analysis for interpreting co-culture-associated regulatory changes. We also developed DeepCluster ICA as a scalable post-ICA consensus framework. Rather than altering linear ICA itself, this approach improves component aggregation across repeated decompositions by combining latent embedding with probabilistic clustering.^34^^,^^35^^,^^36^^,^^37^ In our dataset, DeepCluster ICA produced module structures broadly consistent with optICA while reducing computational burden.

Previous research on microalgal circadian rhythms has mainly utilized axenic cultures, investigating how external conditions such as light regimes,^4^^,^^12^ nutrient levels,^59^^,^^60^ and metal stress^9^^,^^13^^,^^61^ influence rhythmic gene expression and metabolic cycles. In addition to these external influences, some studies have also examined the role of internal regulatory mechanisms, such as glutathione redox potential, transcription factors, and photoreceptors, in modulating microalgal rhythmic patterns.^14^^,^^15^^,^^16^^,^^17^^,^^45^ However, although related studies have shown that heterotrophic interactions can alter transcriptome dynamics and periodicity in photosynthetic microbes, comparable analyses focused on rhythmic organization in defined diatom-bacterium systems remain limited.^62^^,^^63^ Conversely, studies in higher organisms, including plants and animals, have revealed that host circadian systems can both shape and be modulated by associated microbial communities, affecting nutrient cycling, stress responses, and overall ecosystem functioning.^64^ For instance, plant research has shown how host rhythms regulate the structure and function of rhizosphere microbiota, while animal studies have highlighted intricate host-microbe diel interactions in symbiotic and gastrointestinal environments.^65^ By examining co-culture-associated effects on diel transcriptomic organization in a defined diatom-bacterium system, our study provides a tractable framework for investigating how bacterial presence may influence microalgal rhythmic regulation under controlled laboratory conditions.

Our results suggest that prolonged co-culture can be associated with substantial disruption of diel transcriptomic organization and physiological performance in a model diatom. Rather than supporting a single-factor explanation, the data are more consistent with a late-stage co-culture state shaped by multiple interacting influences, including bacterial interaction, nutrient depletion, and culture-environmental changes. From a broader perspective, these findings highlight the importance of considering diel dynamics when interpreting algal responses in co-culture systems and when designing more stable algal-bacterial consortia for future experimental or biotechnological applications. Recent studies have shown that differences in metabolic activity and light-driven rhythms between autotrophic and heterotrophic partners can affect the robustness, productivity, and long-term stability of synthetic communities.^66^^,^^67^^,^^68^ Optimizing such consortia may therefore benefit from balancing resource exchange and division of labor, tuning temporal regulation and photoperiod compatibility, and minimizing destabilizing effects associated with asynchronous growth or metabolic interactions.

In summary, our integrated systems approach captures the temporal progression of the *P. tricornutum*-*A. fischeri* consortium, from an early co-culture state to a later phase characterized by competitive stress, declining photosynthetic performance, and altered diel coordination of metabolism and gene expression. Under prolonged bacterial coexistence, *P. tricornutum* shifts away from growth optimization toward a more stress-associated metabolic state. Beyond delineating the instability features of this model diatom-bacterium system, our study establishes a practical framework that integrates controlled co-culture, genome-scale modeling, and AI-assisted transcriptomics to interrogate how bacterial association shapes diel physiology. In doing so, this work advances current understanding of phytoplankton chronobiology and provides a foundation for future investigations of rhythmic regulation and stress dynamics in algal-bacterial systems.

### Limitations of the study

Several limitations should be acknowledged. First, the study was conducted in a simplified two-species laboratory co-culture and thus does not capture the full complexity of natural microbial communities associated with phytoplankton. Second, the lack of a matched axenic transcriptomic control at day 8 precludes the strict attribution of the late-stage transcriptional differences specifically to bacterial interaction. Third, the current experimental design also does not allow the relative contributions of bacterial interaction, nutrient depletion, culture environment changes, and algal growth phase to be quantitatively disentangled. Fourth, the study focused primarily on diatom responses, without bacterial transcriptomics or direct measurements of interspecies metabolite exchange. Finally, the GEM/FBA results should be interpreted with caution, as they are model-based predictions rather than empirical measurements.

Notwithstanding these limitations, the present dataset and analytical workflow offer a valuable foundation for hypothesis generation in future studies of algal-bacterial systems. The identified iModulons represent candidate regulatory modules, and the transcriptome-informed GEM framework can facilitate the development of more refined dynamic models in complex consortia.^69^

## Resource availability

### Lead contact

Requests for further information and resources should be directed to and will be fulfilled by the lead contact, Weiqi Fu (weiqifu@zju.edu.cn).

### Materials availability

This study did not generate new unique reagents.

### Data and code availability

• Sequencing data have been deposited at NCBI BioProject as PRJNA1280653 (https://www.ncbi.nlm.nih.gov/bioproject/PRJNA1280653) and PRJNA1171500 (https://www.ncbi.nlm.nih.gov/bioproject/PRJNA1171500) and are publicly available as of the date of publication.
• All original code has been deposited at GitHub and is publicly available as of the date of publication at: https://github.com/Gusicun/DeepCluster_ICA/tree/main.
• Any additional information required to reanalyze the data reported in this paper is available from the lead contact, Weiqi Fu (weiqifu@zju.edu.cn), upon request.

## Acknowledgments

This study was supported by the 10.13039/501100001809National Natural Science Foundation of China Joint Fund Project (grant no. U23A2034), Zhejiang Province Leading Geese Plan (grant no. 2024C03110), and the Hundred Talents Program of Zhejiang University. We are grateful to OE Biotech, Inc. (Shanghai, China) for their assistance in sequencing and bioinformatics analysis of the RNA libraries. We also acknowledge the Instrumentation Platform of the Ocean College, Zhejiang University, for their support in conducting the SEM experiments. Additionally, we sincerely thank the anonymous reviewers for their constructive comments.

## Author contributions

Conceptualization, W.F., B.O.P., and Y.Y.; data curation, J.C. and H.D.; formal analysis, J.C. and H.D.; funding acquisition, W.F. and F.Z.; investigation, J.C. and Y.S.; methodology, J.C., H.D., and Y.S.; project administration, W.F. and F.Z.; resources, W.F. and F.Z.; software, H.D.; supervision, W.F. and F.Z.; validation, J.C. and H.D.; visualization, J.C. and H.D.; writing – original draft, J.C. and H.D.; writing – review and editing, J.C., H.D., Y.S., Y.Y., M.K., F.Z., H.Z., K.Z., B.O.P., and W.F. J.C. and H.D. contributed equally to this work and are recognized as co-first authors.

## Declaration of interests

The authors declare no competing interests.

## Declaration of generative AI and AI-assisted technologies in the writing process

During the preparation of this work, the author(s) used DeepSeek V4-Flash, an artificial intelligence-assisted tool, exclusively for grammar and spelling checks. The tool was not used for content generation, scientific writing, or any aspect of data analysis or interpretation. After using this tool, the author(s) reviewed and edited the content as needed and take(s) full responsibility for the content of the publication.

## STAR★Methods

### Key resources table

**Table:** 

REAGENT or RESOURCE	SOURCE	IDENTIFIER
**Bacterial and virus strains**		

*Aliivibrio fischeri*	Marine Culture Collection of China	MCCC 1H00019

**Chemicals, peptides, and recombinant proteins**		

Triton X-100	Solarbio	Cat# T8200
Penicillin-Streptomycin-Gentamicin solution	Solarbio	Cat# P1410
DAPI	Thermo Fisher Scientific	Cat# D1306
Glutaraldehyde solution	Sigma-Aldrich	Cat# G6257
TRIzol reagent	Invitrogen	Cat# 15596026
Bovine serum albumin	Solarbio	Cat# A8020
Salmon sperm DNA	Solarbio	Cat# D8080

**Critical commercial assays**		

Polysaccharide Assay Kit	Solarbio	Cat# BC0030
Bradford Protein Assay Kit	Biosharp	Cat# BL524A
VAHTS Universal V6 RNA-seq Library Prep Kit	Vazyme	Cat# NR604

**Deposited data**		

RNA-seq data	NCBI BioProject	PRJNA1280653; https://www.ncbi.nlm.nih.gov/bioproject/PRJNA1280653
RNA-seq data	NCBI BioProject	PRJNA1171500; https://www.ncbi.nlm.nih.gov/bioproject/PRJNA1171500
DeepCluster ICA source code	GitHub	https://github.com/Gusicun/DeepCluster_ICA/tree/main

**Experimental models: Organisms/strains**		

*Phaeodactylum tricornutum*	Culture Collection of Algae and Protozoa	CCAP 1055/1

**Software and algorithms**		

Python	Python Software Foundation	Version 3.13.11
NumPy	NumPy developers	Version 2.1.3
Pandas	pandas development team	Version 2.2.3
scikit-learn	scikit-learn developers	Version 1.6.1
SciPy	SciPy developers	Version 1.15.3
Matplotlib	Matplotlib development team	Version 3.10.0
PyTorch	PyTorch Foundation	Version 2.12.1+cpu
DeepCluster ICA	This paper	https://github.com/Gusicun/DeepCluster_ICA/tree/main
Fastp	OpenGene	Version 0.23.4
HISAT2	Johns Hopkins University CCB	Version 2.2.1
HTSeq	HTSeq developers	Version 2.0.5
DESeq2	Bioconductor	Version 1.44.0
R	R Foundation for Statistical Computing	Version 4.4.1
MetaCycle	Bioconductor	Version 1.2.0
JTK_CYCLE	Implemented in MetaCycle	MetaCycle version 1.2.0
ARSER	Implemented in MetaCycle	MetaCycle version 1.2.0
g:Profiler	University of Tartu	https://biit.cs.ut.ee/gprofiler
Bioladder	Bioladder	https://www.bioladder.cn
MATLAB	MathWorks	Release R2025a
COBRA Toolbox	OpenCOBRA Project	https://github.com/opencobra/cobratoolbox
Gurobi Optimizer	Gurobi Optimization	Version 12.0.3
iModulonMiner optICA pipeline	iModulonMiner	Sastry et al.,^33^^,^^35^ McConn et al.,^34^ and Catoiu et al.^70^
Microsoft Excel	Microsoft	Microsoft Excel for Microsoft 365
Gurobi Optimizer	Gurobi Optimization	Version 12.0.3

**Other**		

AquanPen	Photon Systems Instruments	Model AP110/C
Microplate reader	Agilent BioTek	Synergy H1
Scanning electron microscope	Carl Zeiss	Sigma 500
Spectrophotometer	Thermo Scientific	NanoDrop 2000
Bioanalyzer	Agilent Technologies	Agilent 2100 Bioanalyzer
RNA-sequencing platform	Illumina	NovaSeq 6000

### Experimental model and study participant details

#### *Phaeodactylum tricornutum*

Axenic *Phaeodactylum tricornutum* CCAP 1055/1 was obtained from the Culture Collection of Algae and Protozoa (CCAP), Scotland, UK. Cultures were maintained at 20°C in enriched artificial seawater (EASW) medium. Additional cultivation conditions used for each experimental group are described in the method details section. Sex does not apply to this experimental model.

#### Aliivibrio fischeri

*Aliivibrio fischeri* MCCC 1H00019 was obtained from the Marine Culture Collection of China and maintained at 20°C in marine broth 2216 medium. The bacterial strain was co-cultured with *P. tricornutum* under the conditions described in the method details section. Sex does not apply to this experimental model.

### Method details

#### Culture conditions

Axenic culture of *Phaeodactylum tricornutum* CCAP 1055/1 was purchased from the Culture Collection of Algae & Protozoa (CCAP), Scotland, UK, and cultured at 20 °C using enriched artificial seawater (EASW) medium. *Aliivibrio fischeri* (MCCC 1H00019) was obtained from the Marine Culture Collection of China and cultured at 20 °C using marine broth 2216 medium.

Based on a previously reported protocol, *P. tricornutum* was axenized by treating surface-attached bacteria with 20 μg/mL Triton X-100, followed by separation using a 10 μm filter.^71^ The culture was then passaged at low density (∼1×10^5^ cells/mL) in an EASW medium containing a 1:100 dilution of Penicillin-Streptomycin-Gentamicin solution. Prior to the algal-bacteria co-culture experiment, the algal culture was washed with sterile EASW medium to remove antibiotics. DAPI staining combined with fluorescence microscopy showed no bacterial signal in the control or HAC cultures, whereas bacterial signals were observed in the co-culture groups (Figure S18).

#### Experimental design for algal-bacteria co-culture

This study was designed to investigate the rhythmic gene expression changes of *P. tricornutum* over 24 h in batch culture. Four transcriptomic groups were included: Control, HAC, Co4d, and Co8d. The two co-culture groups correspond to the exponential growth phase (day 4, Co4d) and the stationary phase (day 8, Co8d), respectively. The co-culture system was cultured at 20 ± 1°C under white light with a photosynthetically active radiation intensity of 100 μmol photons·m^−2·^s^−1^ and stirred at 200 rpm in a 12-h light/12-h dark cycle. A co-culture medium was established by supplementing EASW with 30% (*v/v*) marine broth to support both diatoms and bacterial growth, based on experiments (Figure 1C) that omitted or used excess marine broth slowed *P. tricornutum* growth.

The initial cell densities of *P. tricornutum* and *A. fischeri* were set as 1×10^6^ cells/mL and 4×10^4^ cells/mL, respectively. For the heterotrophic/autotrophic axenic culture (HAC) group, *P. tricornutum* was cultivated alone in EASW supplemented with 30% marine broth under the same 12 h:12 h light-dark cycle and at the same initial cell density as in the co-culture system, but without the addition of *A. fischeri*. As a comparison (Control), *P. tricornutum* was maintained in an EASW medium without the marine broth mixture.

Maximum quantum yield (QY) was measured using an AquanPen (AP110/C, PSI, Czech Republic). Due to difficulties in counting microalgae within algal-bacteria aggregates using a hemocytometer, algal cell density was estimated via chlorophyll fluorescence (excitation 488 nm, emission 695 nm) intensity using a microplate reader (Synergy H1, Agilent BioTek, Beijing, CHN). The cell density of bacteria was determined by CFU counts from dilution plating.

Pigments were extracted from cells on 0.45 μm filters using 100% methanol at −20°C for 24 h. Absorbance at 632, 652, 665, 750, and 480 nm quantified chlorophyll *a*, chlorophyll *c*, and carotenoids.^72^ The pigment-to-dry weight ratio was calculated using the linear relationship: DW = 8.06 × 10^−5^ × chlorophyll fluorescence.

Nitrate nitrogen (NO_3_-N) and orthophosphate (PO_4_-P) were measured from filtrates obtained via 0.45 μm glass fiber filters. NO_3_-N was assessed at 220 nm with 275 nm correction for organic matter interference, while PO_4_-P was quantified at 700 nm using the phosphomolybdenum blue method with potassium antimonyl tartrate as a catalyst.

The measurement of EPS was primarily based on the method by Ren et al. (2024).^73^ 5 mL of the algae-bacteria co-culture was filtered through a 0.45 μm membrane, washed twice with deionized water, and subjected to a 30-min water bath at 55°C. The mixture was then centrifuged, and the supernatant was used to measure polysaccharide, protein, and DNA content. Polysaccharides were measured using the BC0030 Kit (Solarbio, Beijing, CHN). Proteins were determined using the BL524A Bradford Protein Assay Kit with Bovine Serum Albumin as the standard (Biosharp, Hefei, CHN). DNA was determined using the diphenylamine method with salmon sperm DNA as the standard (Solarbio, Beijing, CHN). The total EPS concentration was calculated as the sum of polysaccharides, proteins, and DNA. The COD equivalents were 1.4 mg COD per mg of Bovine Serum Albumin, 1.07 mg COD per mg of glucose, and 0.74 mg COD per mg of DNA.^73^

All physiological parameters, including QY, chlorophyll fluorescence, bacterial cell density, chlorophyll *a*, chlorophyll *c*, carotenoids, NO_3_-N, PO_4_-P, and EPS content (polysaccharides, proteins, and DNA), were measured in triplicates.

#### Scanning electron microscope for algae-bacteria co-culture samples

Algae-bacteria co-culture was fixed in a 2.5% (v/v) glutaraldehyde solution at 4°C overnight and then filtered through a 3 μm membrane (N66, Haining Yibo) to retain the cells. The membrane underwent gradient dehydration with ethanol concentrations of 0%, 20%, 40%, 60%, 80%, and 100%, followed by natural drying overnight at room temperature. The dried membrane was sputter-coated with gold and observed using an SEM (Sigma 500, Carl Zeiss, UK) to document cell structure and surface details.

#### Transcriptome sampling of rhythmic algae-bacteria co-culture under day-night light conditions

For transcriptomic analysis, samples were collected every 4 h over a 24-h cycle at seven zeitgeber time points (ZT1, ZT5, ZT9, ZT13, ZT17, ZT21, and ZT25). Co4d and Co8d were sampled on days 4–5 and 8–9, respectively, while Control and HAC were sampled at the same time points on days 4–5 only. During cell harvesting, co-culture samples were passed through a 10 μm filter, resuspended in sterile seawater, and refiltered three times before final collection by centrifugation. This step was used as a coarse washing/enrichment procedure to reduce bacterial carryover prior to RNA extraction, rather than as a strict size-based separation of *P. tricornutum*. Because RNA-seq libraries were prepared using poly(A)-enriched RNA, the contribution of bacterial transcripts to the final dataset was expected to be substantially reduced. For all groups, three biological replicates were taken at each time point.

#### RNA extraction, sequencing, and bioinformatics analysis protocols

Total RNA was isolated using TRIzol reagent following the manufacturer’s protocol. RNA purity and concentration were measured with a NanoDrop 2000 spectrophotometer (Thermo Scientific, USA), while RNA integrity was evaluated using the Agilent 2100 Bioanalyzer (Agilent Technologies, Santa Clara, CA, USA). The transcriptome libraries were prepared using the VAHTS Universal V6 RNA-seq Library Prep Kit, following the instructions provided.

RNA sequencing was performed on the libraries using the Illumina Novaseq 6000 platform, generating 150 bp paired-end reads. Each sample yielded approximately 60M raw reads, totaling around 9G of raw bases. The fastp software was used to process raw reads in fastq format, filtering out low-quality reads to obtain clean reads for subsequent analyses. HISAT2 was employed for genome alignment, and gene expression levels were quantified as FPKM values. HTSeq-count was then used to obtain read counts for each gene. Differential gene expression analysis was conducted using DESeq2. Genes with a q-value <0.05 and a fold change >2 or <0.5 were considered differentially expressed genes (DEGs).

#### Identification and enrichment analysis of rhythmic genes

Rhythmic transcripts were identified using JTK_CYCLE implemented in MetaCycle, a widely used method for detecting periodic signals in genome-scale time-series data.^29^^,^^74^^,^^75^ The FPKM values for each sample were converted to TPM for the subsequent identification of rhythmic genes. TPM values less than 2 were filtered, leaving 10,815 genes. For downstream rhythmicity analysis and visualization, TPM values were first averaged across biological replicates at each condition and time point. Each gene was then min–max normalized across all samples. Using the MetaCycle package (v1.2.0) in R (v4.4.1), rhythmic genes with a q-value less than 0.05 and period >0 were identified through the Jonckheere-Terpstra-Kendall (JTK) algorithm.^29^^,^^74^ The ARSER algorithm was also used to identify rhythmic genes for comparison with the JTK results. GO and KEGG enrichment analyses of the rhythmic genes were conducted online using g:Profiler,^76^ and results were visualized with the R package (v4.4.1) and Bioladder (bioladder.cn).^77^ A Tau index was further calculated for each gene in each group from the log_2_(TPM +1) values across the seven zeitgeber time points. Higher Tau values indicate stronger temporal specificity. Differences between rhythmic and non-rhythmic genes were tested using two-sided Wilcoxon rank-sum tests.

#### Integration of transcriptomic data with genome-scale metabolic modeling

To infer condition- and time-dependent changes in metabolic state, genome-scale metabolic modeling was performed in MATLAB R2025a using the COBRA Toolbox (2025) and the Gurobi (v12.0.3) linear programming solver, with the published SBML model iLB1027_lipid for *P. tricornutum*.^30^^,^^31^^,^^32^^,^^54^^,^^78^^,^^79^ For each experimental condition, transcriptomic TPM values were mapped to the corresponding genes in the metabolic model. Gene expression levels were then proportionally used to constrain reaction flux bounds based on gene-protein-reaction (GPR) associations, following established COBRA Toolbox workflows.^78^ Flux balance analysis (FBA) was conducted to obtain biomass production rates and reaction flux distributions.^32^ Daytime and nighttime periods were defined according to zeitgeber time (ZT), with daytime samples collected at ZT1, ZT5, ZT9, and ZT13, and nighttime samples collected at ZT13, ZT17, ZT21, and ZT25.

#### Baseline robust independent component analysis using iModulonMiner

As a baseline approach for transcriptome decomposition, we applied the established iModulonMiner optICA pipeline.^33^^,^^34^^,^^35^^,^^70^ Within this pipeline, independent component analysis (ICA) was performed using the FastICA implementation from scikit-learn.

The log-transformed Transcripts Per Million (log-TPM) expression matrix (10,815 genes × 213 samples), including three biological replicates, was decomposed 100 times with distinct random seeds, utilizing a maximum of 100 iterations and a convergence tolerance of 10^−7^. For each given dimensionality, robust ICs were identified by performing pairwise comparisons of the resulting gene-weight vectors (ICs), treating a component and its sign-flipped counterpart as identical if their absolute Pearson correlation coefficient exceeded a certain threshold. These components were then clustered using DBSCAN (maximum distance = 0.1; minimum core size = 50).

To determine the optimal ICA dimensionality, this entire robust IC identification procedure was iterated across candidate dimensions from d = 50 to d = 200, in increments of 10. For each candidate dimensionality, we quantified two key metrics for the identified robust ICs: first, single-gene dominated ICs, defined as those where the absolute weight of the highest-weighted gene was more than twice that of the second-highest-weighted gene; and second, ICs highly similar to components from the largest-tested dimension (d = 200), identified if their absolute cosine similarity with any reference component exceeded 0.7. The optimal dimensionality was then selected as the smallest d where the count of robust multi-gene ICs (i.e., total robust ICs minus single-gene dominated ICs) was greater than or equal to the count of robust ICs highly similar to those from the largest-tested dimension. Applying this criterion, the optimal dimensionality was determined to be d = 110, yielding a final set of 72 robust independent components.

#### Iterative ICA and component harvesting

To improve the scalability and robustness of component harvesting across repeated ICA runs, we developed DeepCluster ICA, an AI-assisted post-ICA consensus framework. DeepCluster ICA retains the linear FastICA decomposition step and replaces conventional all-against-all similarity clustering with latent embedding and probabilistic clustering for component aggregation across random restarts.^34^^,^^35^^,^^36^^,^^37^^,^^38^^,^^70^ Before aggregation, the ICA dimensionality was chosen by a restart-based sweep over a grid of candidate component numbers. Similar to optICA, we selected the smallest that balanced component recoverability against over-decomposition and fixed this dimensionality for all subsequent analyses. Initial decomposition of the log-TPM expression matrix (10,815 genes × 213 samples) was performed using the FastICA implementation from scikit-learn, run 100 times at the selected with distinct random seeds.

To account for the non-deterministic nature of ICA, the decomposition was repeated across 100 random initializations, with each run using a maximum of 100 iterations and a convergence tolerance of 1 × 10^−7^. Each FastICA run yielded a set of signed gene-weight vectors (*M*) and the corresponding sample-activation vectors (*A*). Crucially, DeepCluster ICA diverges from methods that rely on explicit component-wise correlation calculations. Instead, all gene-weight vectors obtained across FastICA restarts were pooled into a single matrix, denoted as *M*_flat_, with *K* rows and *G* columns, where *K* is the total number of collected components and *G* is the number of genes. To place every gene on a comparable scale, each gene’s weights across all *K* components were independently z-scored, forming the standardized matrix *M*_norm_. These normalized vectors serve as input to a deep AutoEncoder (AE), which performs non-linear dimensionality reduction and feature learning. The AE consisted of an encoder and a decoder, with multiple hidden layers (e.g., 512 and 256 units) and ReLU activation. The encoder mapped each high-dimensional gene-weight vector to a lower-dimensional latent representation, and the decoder reconstructed the original input from that representation.

The AE was pre-trained to minimize the mean squared reconstruction error:

$$L_{AE}=\frac{1}{K}\sum_{i=1}^{K}{\left\|M_{norm,i}-D\left(E\left(M_{norm,i}\right)\right)\right\|}_{2}^{2}$$

L1 regularization was further applied to the weights of sparse linear layers to promote feature sparsity. The learned latent representations were then clustered using a Bayesian Gaussian Mixture Model (BGMM). Unlike a conventional GMM, the BGMM was specified with a Dirichlet Process prior on component weights. This allowed the model to infer the effective number of clusters up to a predefined maximum (e.g., 200), thereby reducing the need for manual cluster-number selection as in methods such as DBSCAN. The BGMM modeled the latent space as a mixture of Gaussian components and assigned each latent representation to a cluster based on posterior probabilities.

#### Robust component identification

This design defines robust components based on both clustering consistency and reproducibility across ICA restarts. Robust independent components are identified through a two-stage filtering process applied to the BGMM clusters. For each cluster *c*, we recorded the set of assigned components, the BGMM mixture weight of the cluster, and the set of distinct FastICA runs represented within that cluster. A cluster was considered robust if it passed both the BGMM weight filter and the multi-run reproducibility filter. Specifically, the cluster weight had to exceed a predefined threshold, and the cluster had to be recovered from at least a minimum fraction of all FastICA runs. This ensures that robust clusters are both statistically significant and consistently discovered across a substantial portion of the stochastic ICA decompositions.

For each robust cluster, the corresponding gene-weight vectors and sample-activation vectors were collected. To ensure a consistent orientation, vectors showing a negative Pearson correlation with the reference vector were sign-flipped before averaging. The consensus gene-weight vector and sample-activation vector for each robust cluster were then calculated as the element-wise mean of the aligned vectors.

### Quantification and statistical analysis

Statistical analyses of physiological measurements were performed using Microsoft Excel for Microsoft 365. Unless otherwise stated, quantitative data are presented as the mean ± SD of three biological replicates (*n* = 3), where n represents independent cultures. Comparisons between two groups were performed using two-tailed unpaired Student’s t-tests. Significance was indicated as ∗*p* < 0.05, ∗∗*p* < 0.01, ∗∗∗*p* < 0.001, and ∗∗∗∗*p* < 0.0001. Rhythmic genes were identified using the Jonckheere–Terpstra–Kendall (JTK) algorithm implemented in MetaCycle, with a *q* value <0.05 and an estimated period >0. Differences in Tau indices between rhythmic and non-rhythmic genes were evaluated using two-sided Wilcoxon rank-sum tests. Exact sample sizes and additional statistical details are provided in the corresponding figure legends and method details. No statistical method was used to predetermine sample size.

### Additional resources

No additional resources are associated with this study.
