# Supplementary material for: Bacterial stress rewires diatom chronobiology and ecosystem function
Source: iScience. 2026 Aug 24;29(9):117307. doi: 10.1016/j.isci.2026.117307 (PMC13524729; doi:10.1016/j.isci.2026.117307)
Supplement: Supplementary file 1 [file mmc1.pdf]

iScience, Volume *29*

Supplemental information

Bacterial stress rewires diatom chronobiology and ecosystem function

Jiwei Chen, Hang Diao, Yixi Su, Yuan Yuan, Manish Kumar, Fan Zhang, Haisheng Zhang, Karsten Zengler, Bernhard O. Palsson, and Weiqi Fu

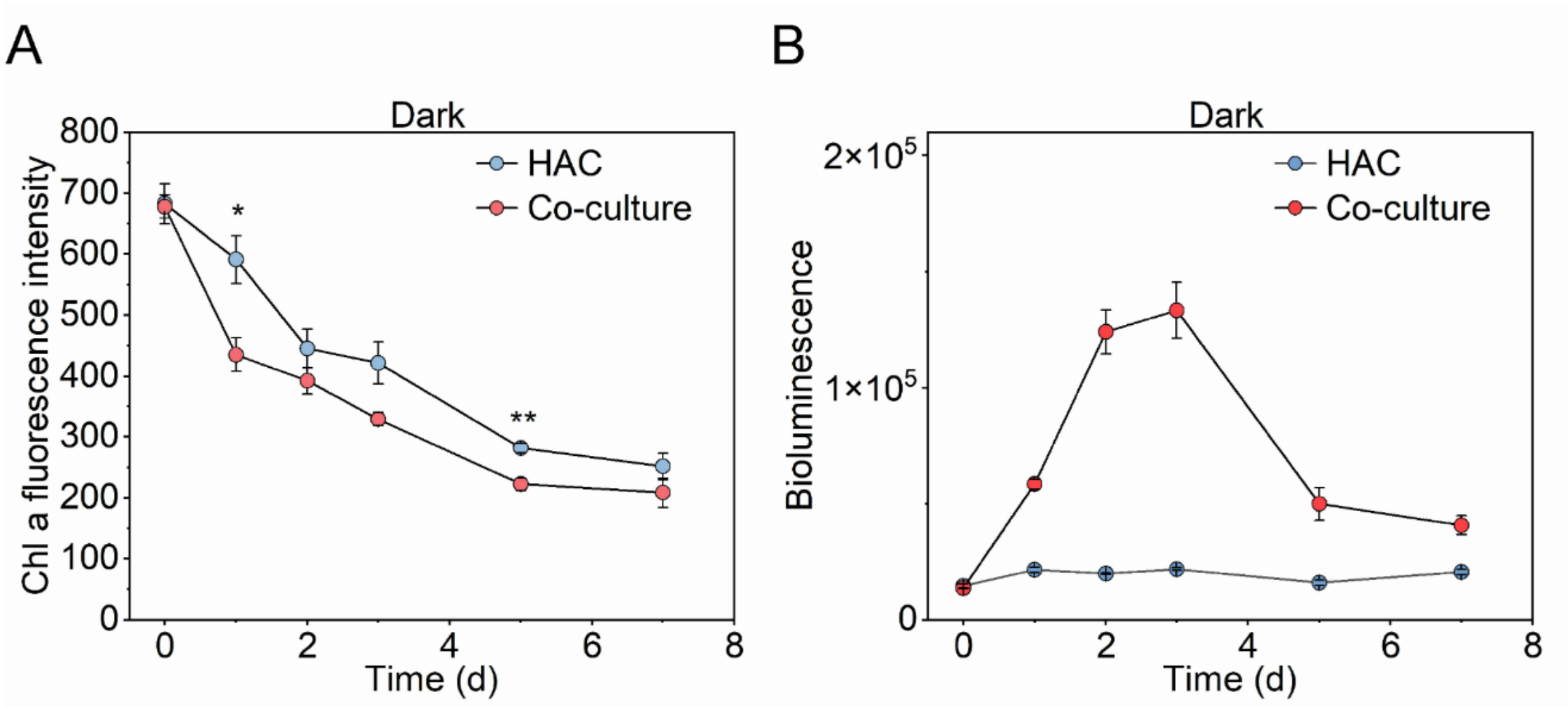

Figure S1. Dynamics of chlorophyll *a* fluorescence intensity (A) and bioluminescence (B) in *P. tricornutum* under dark conditions, comparing co-culture with *A. fischeri* (supplemented with 30% marine broth) to a monoculture control. HAC, heterotrophic/autotrophic axenic culture of *P. tricornutum* on co-culture medium; Co-culture, co-culture of *P. tricornutum* with *A. fischeri* on co-culture medium. Data are presented as mean ± SD from three biological replicates (n = 3), where n represents independent cultures.

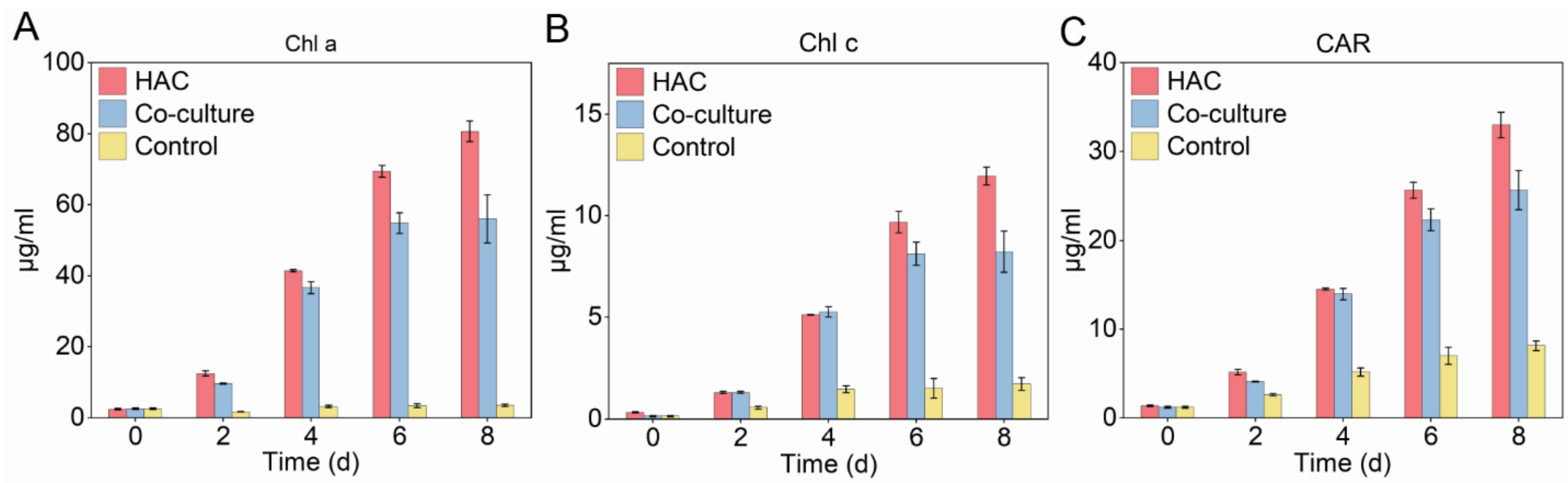

Figure S2. Alterations in photosynthetic pigment composition in response to co-cultivation. (A) Chlorophyll *a* (Chl a); (B) Chlorophyll *c* (Chl c); (C) Total carotenoids (CAR). Experimental conditions were: axenic *P. tricornutum* culture in standard EASW medium (Control), co-culture with *A. fischeri* (Co-culture), and an axenic culture control in co-culture medium (HAC). Control, axenic culture of *P. tricornutum* on EASW medium; HAC, heterotrophic/autotrophic axenic culture of *P. tricornutum* on co-culture medium; Co-culture, co-culture of *P. tricornutum* with *A. fischeri* on co-culture medium. Data are presented as mean ± SD from three biological replicates (n = 3), where n represents independent cultures.

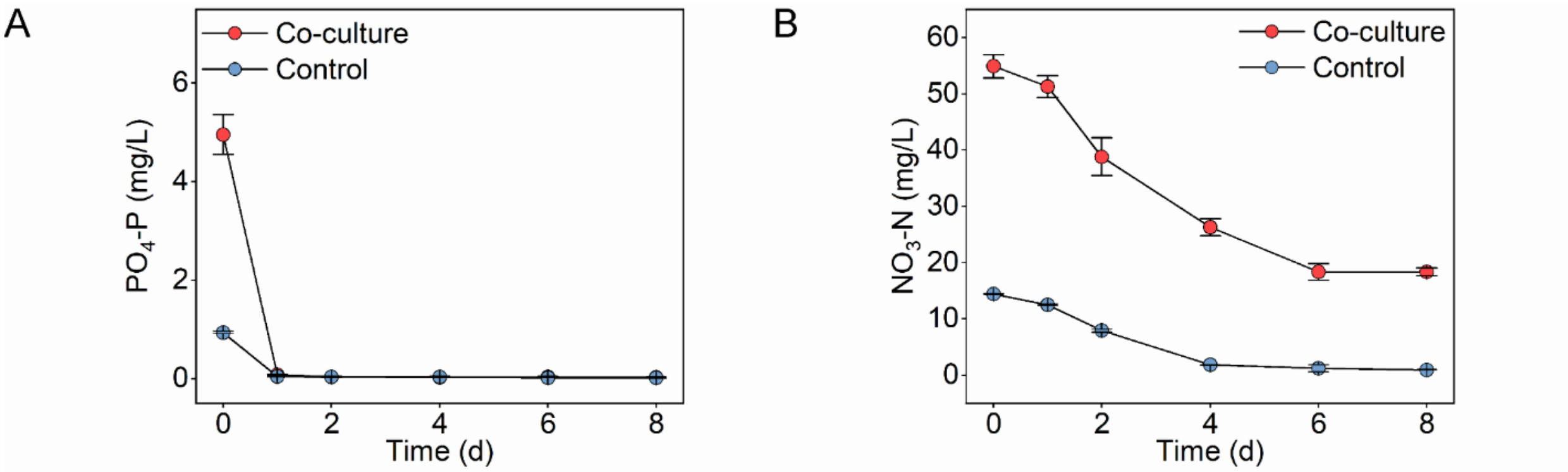

Figure S3. Temporal variations in phosphorus (A) and nitrogen (B) concentrations in co-culture media. Control, axenic culture of *P. tricornutum* on EASW medium; Co-culture, co-culture of *P. tricornutum* with *A. fischeri* on co-culture medium. Data are presented as mean ± SD from three biological replicates (n = 3), where n represents independent cultures.

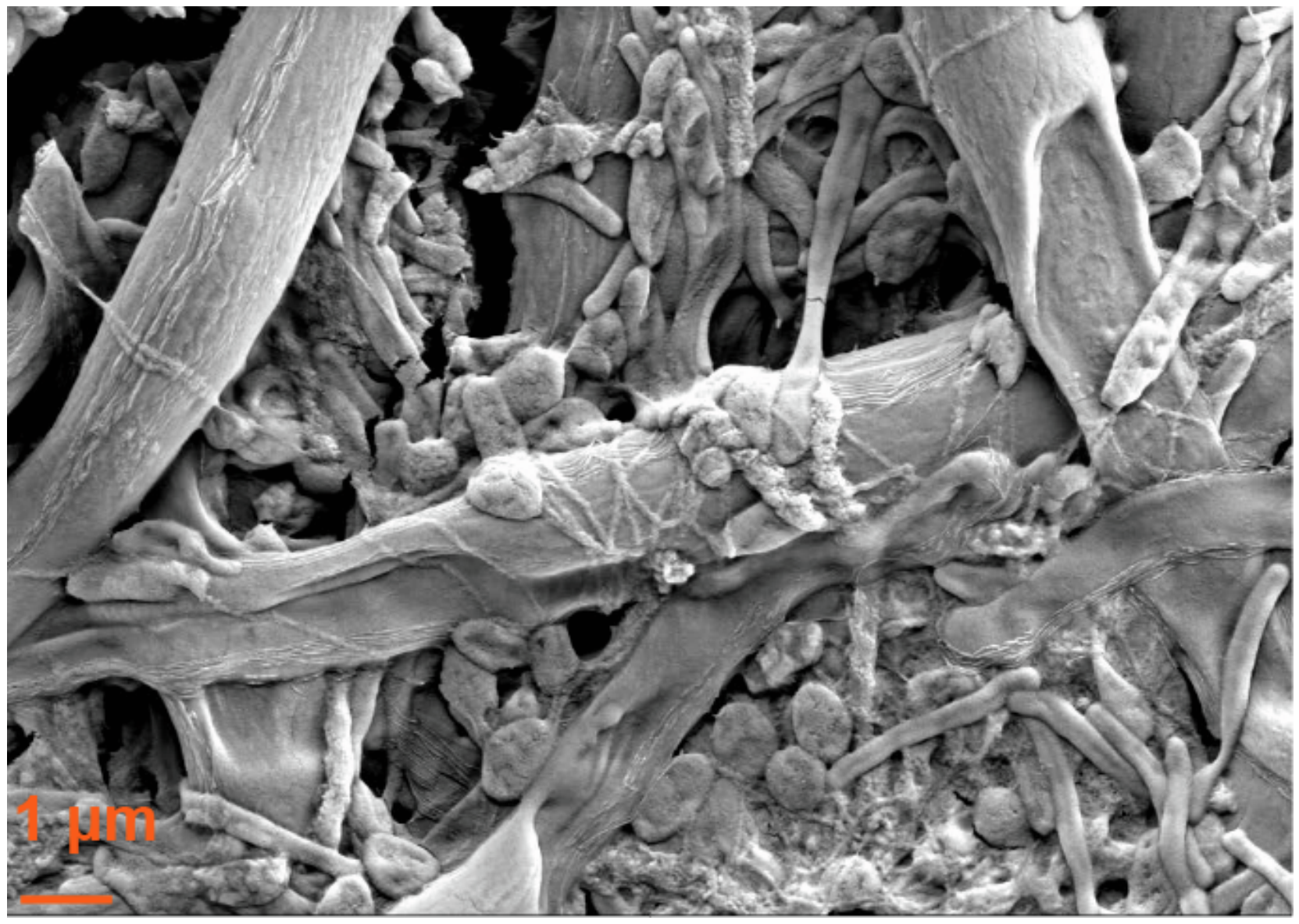

Figure S4. Scanning electron microscopy (SEM) image of the co-culture system, with arrows indicating filamentous EPS secreted by *P. tricornutum* or *A. fischeri* (scale bar: 1 μm).

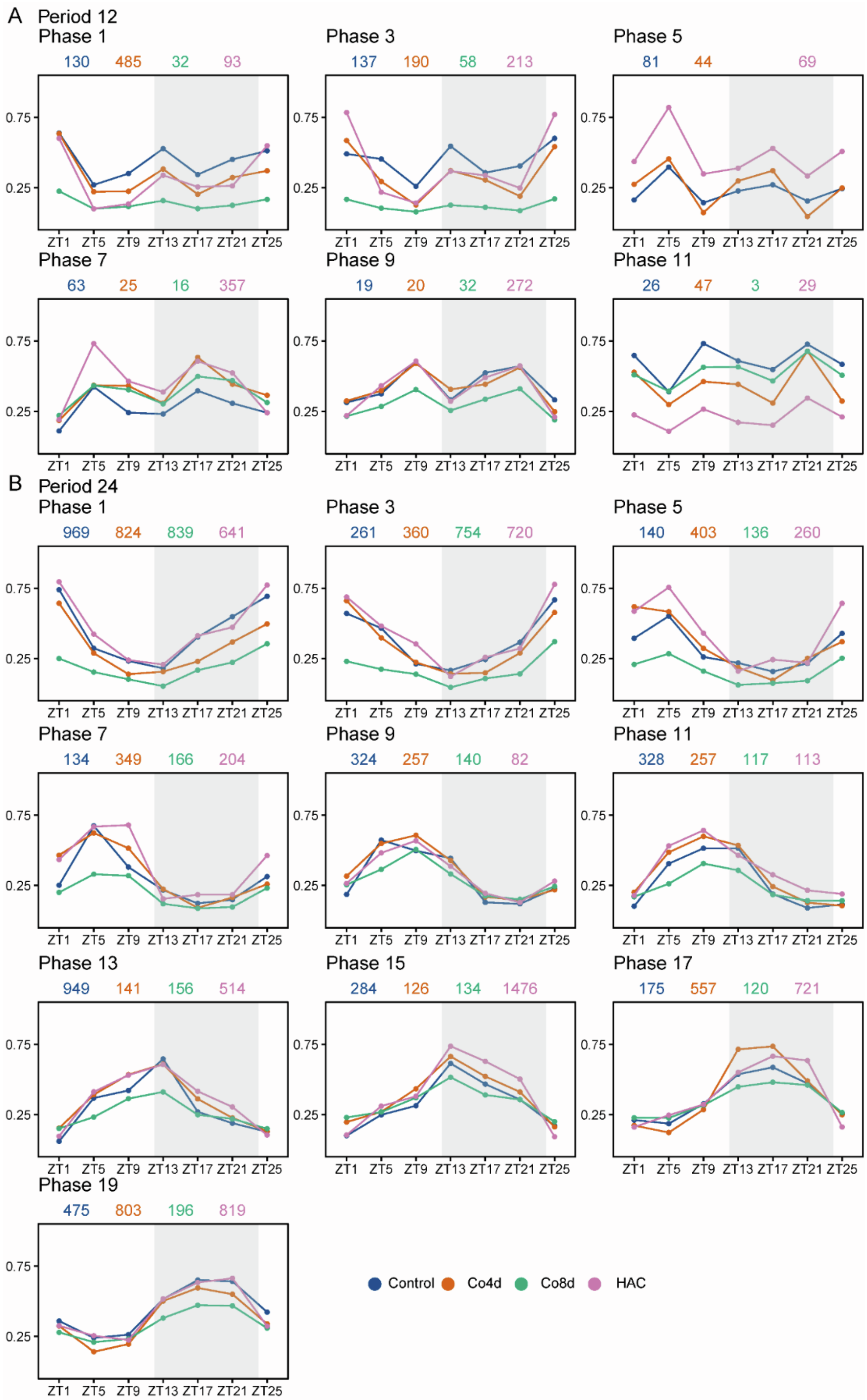

Figure S5. Line plots depicting rhythmic gene counts and expression profiles at all circadian phases over 12 hours (A) and 24 hours (B). Control, axenic culture of *P. tricornutum* on EASW

medium; HAC, heterotrophic/autotrophic axenic culture of *P. tricornutum* on co-culture medium; Co4d, 4-day co-culture of *P. tricornutum* with *A. fischeri* on co-culture medium; Co8d, 8-day co-culture of *P. tricornutum* with *A. fischeri* on co-culture medium.

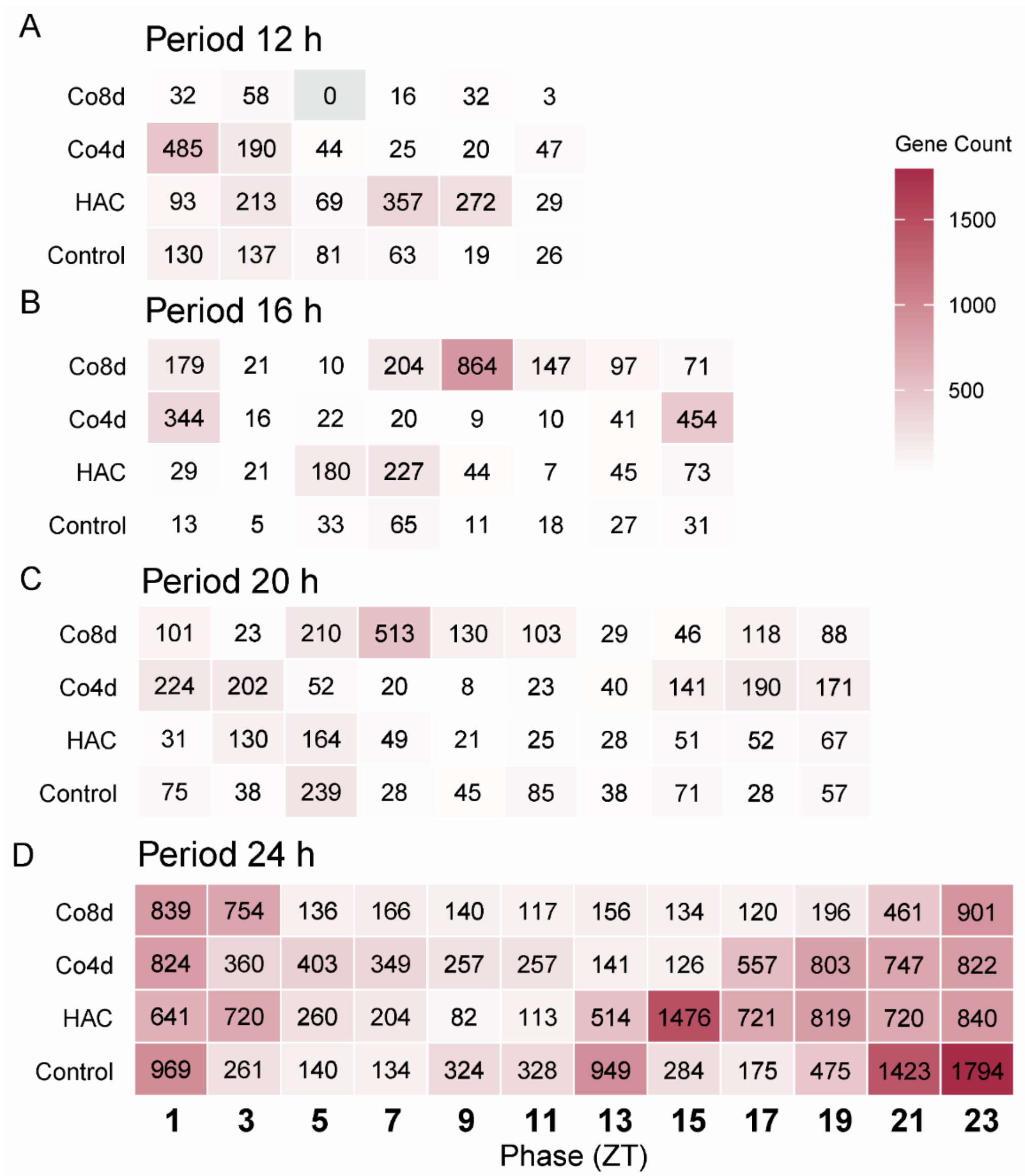

Figure S6. The number of rhythmic genes shared across the four groups (Control, Co4d, Co8d, and HAC groups) at different circadian phases: 12 hours (A), 16 hours (B), 20 hours (C), and 24 hours (D). Control, axenic culture of *P. tricornutum* on EASW medium; HAC, heterotrophic/autotrophic axenic culture of *P. tricornutum* on co-culture medium; Co4d, 4-day co-culture of *P. tricornutum* with *A. fischeri* on co-culture medium; Co8d, 8-day co-culture of *P. tricornutum* with *A. fischeri* on co-culture medium.

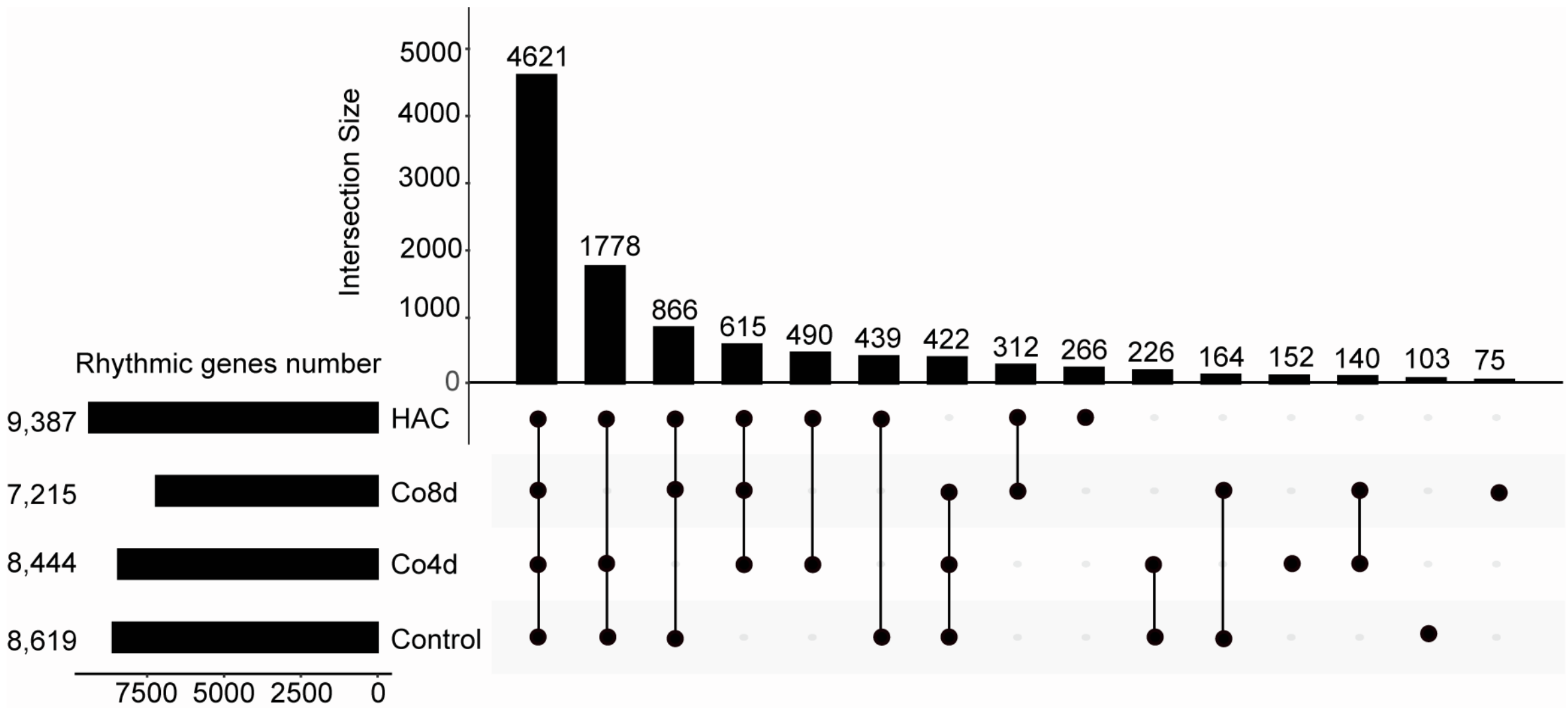

Figure S7. Intersection and specificity of rhythmic genes among the four experimental groups. The UpSet plot shows the total number of rhythmic genes in each group (horizontal bars, left) and the number of genes unique to one group or shared among multiple groups (vertical bars, top). Control, axenic culture of *P. tricornutum* on EASW medium; HAC, heterotrophic/autotrophic axenic culture of *P. tricornutum* on co-culture medium; Co4d, 4-day co-culture of *P. tricornutum* with *A. fischeri* on co-culture medium; Co8d, 8-day co-culture of *P. tricornutum* with *A. fischeri* on co-culture medium.

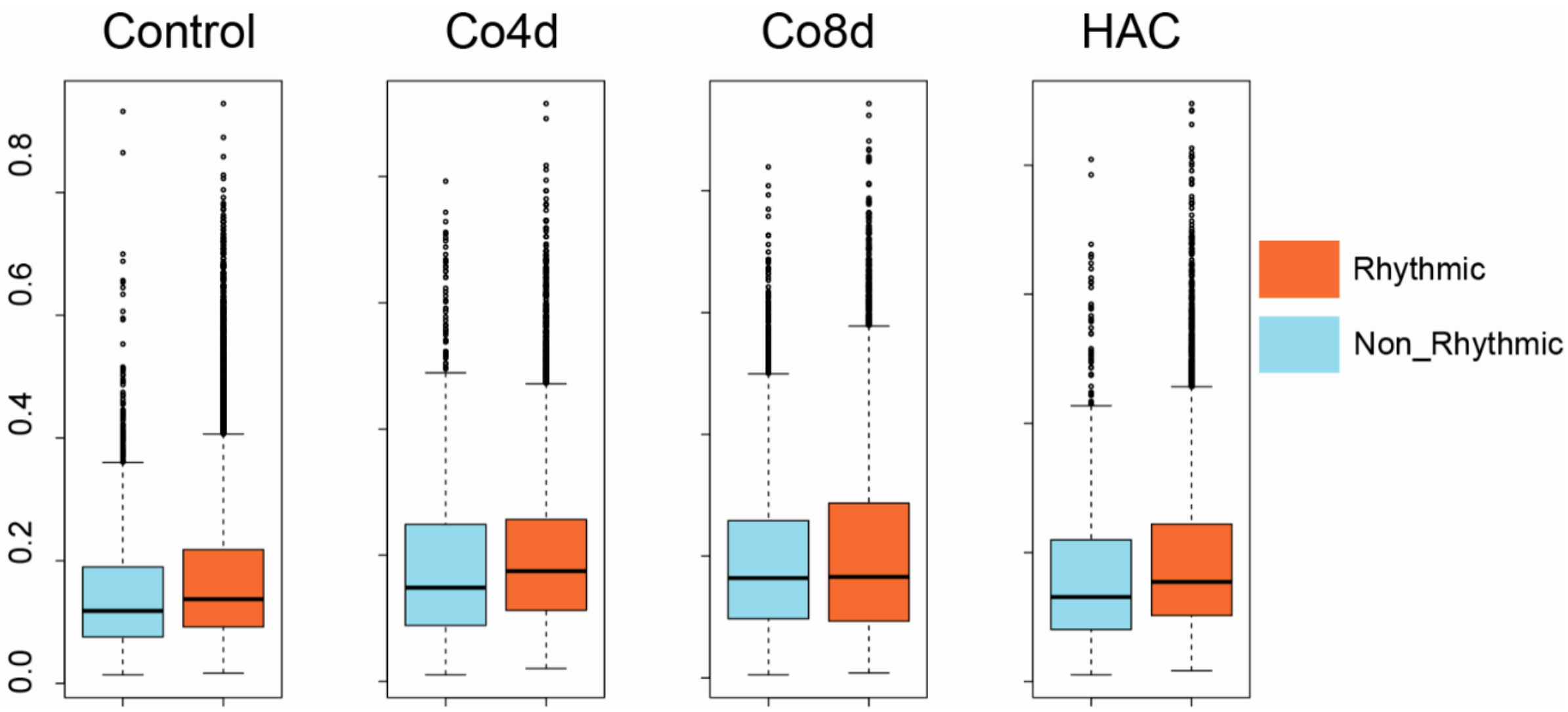

Figure S8. Comparison of circadian rhythmicity strength. Boxplots show the Tau index for genes classified as rhythmic (orange) and non-rhythmic (blue) across all experimental groups. Differences between rhythmic and non-rhythmic genes were tested using two-sided Wilcoxon rank-sum tests. In all conditions, rhythmic genes exhibit a significantly higher Tau index, validating the classification method. Control, axenic culture of *P. tricornutum* on EASW medium; HAC, heterotrophic/autotrophic axenic culture of *P. tricornutum* on co-culture medium; Co4d, 4-day co-culture of *P. tricornutum* with *A. fischeri* on co-culture medium; Co8d, 8-day co-culture of *P. tricornutum* with *A. fischeri* on co-culture medium.

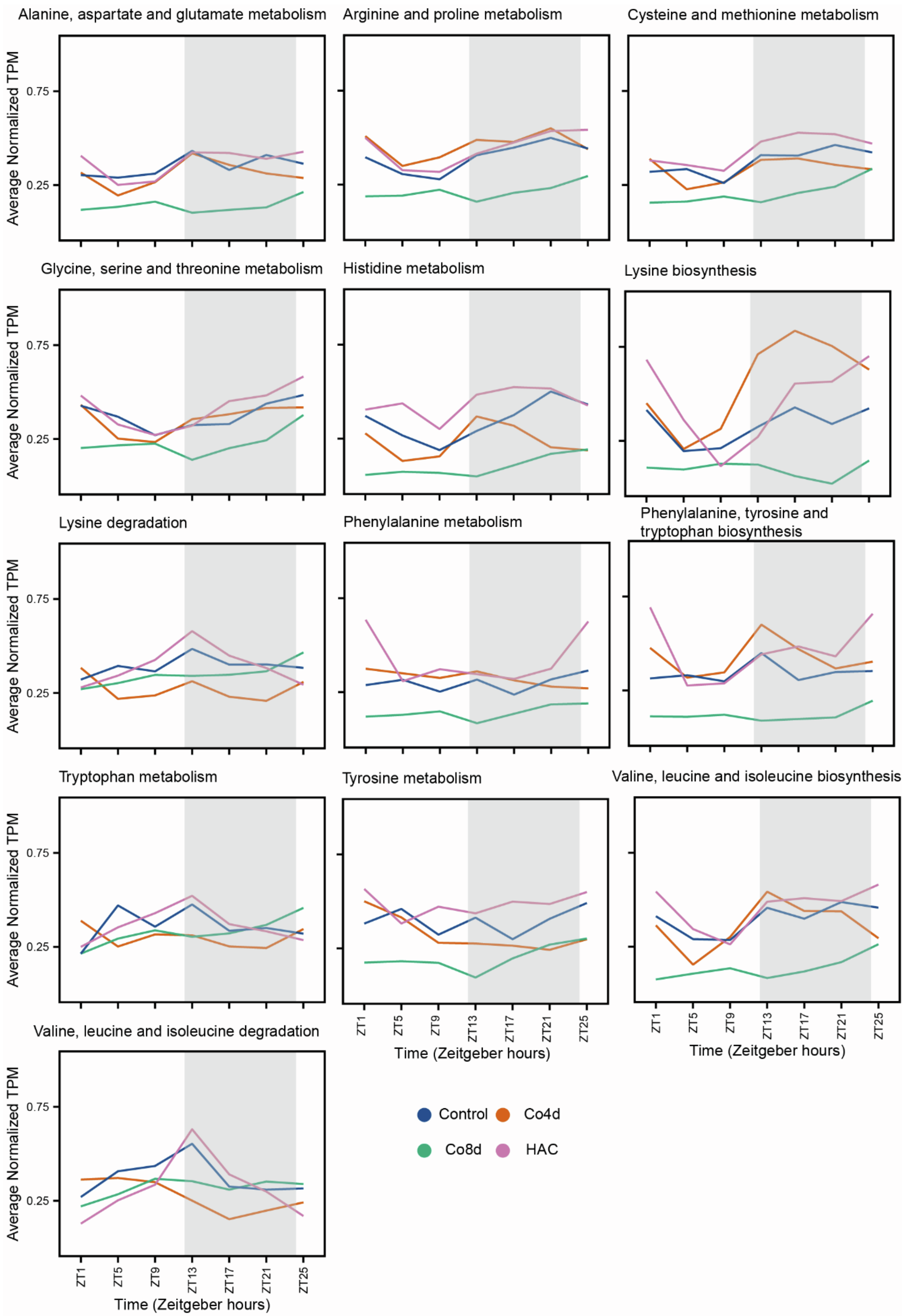

Alanine, aspartate and glutamate metabolism
Arginine and proline metabolism
Cysteine and methionine metabolism
Glycine, serine and threonine metabolism
Histidine metabolism
Lysine biosynthesis
Lysine degradation
Phenylalanine metabolism
Phenylalanine, tyrosine and tryptophan biosynthesis
Tryptophan metabolism
Tyrosine metabolism
Valine, leucine and isoleucine biosynthesis
Valine, leucine and isoleucine degradation
Average Normalized TPM
0.75
0.25
ZT1
ZT5
ZT9
ZT13
ZT17
ZT21
ZT25
Time (Zeitgeber hours)
Control
Co4d
Co8d
HAC

Figure S9. Temporal expression profiles of pathways related to amino acid metabolism. Analysis of time-series transcriptomes showing temporal expression profiles for 13 pathways related to amino acid metabolism. Data are shown for four experimental groups: Control, HAC (heterotrophic axenic culture), Co4d (4-day co-culture), and Co8d (8-day co-culture). The pathways include: Alanine, aspartate and glutamate metabolism; Arginine and proline metabolism; Cysteine and methionine metabolism; Glycine, serine, and threonine metabolism; Histidine metabolism; Lysine biosynthesis; Lysine degradation; Phenylalanine metabolism; Phenylalanine, tyrosine, and tryptophan biosynthesis; Tryptophan metabolism; Tyrosine metabolism; Valine, leucine and isoleucine biosynthesis; and Valine, leucine and isoleucine degradation. Control, axenic culture of *P. tricornutum* on EASW medium; HAC, heterotrophic/autotrophic axenic culture of *P. tricornutum* on co-culture medium; Co4d, 4-day co-culture of *P. tricornutum* with *A. fischeri* on co-culture medium; Co8d, 8-day co-culture of *P. tricornutum* with *A. fischeri* on co-culture medium.

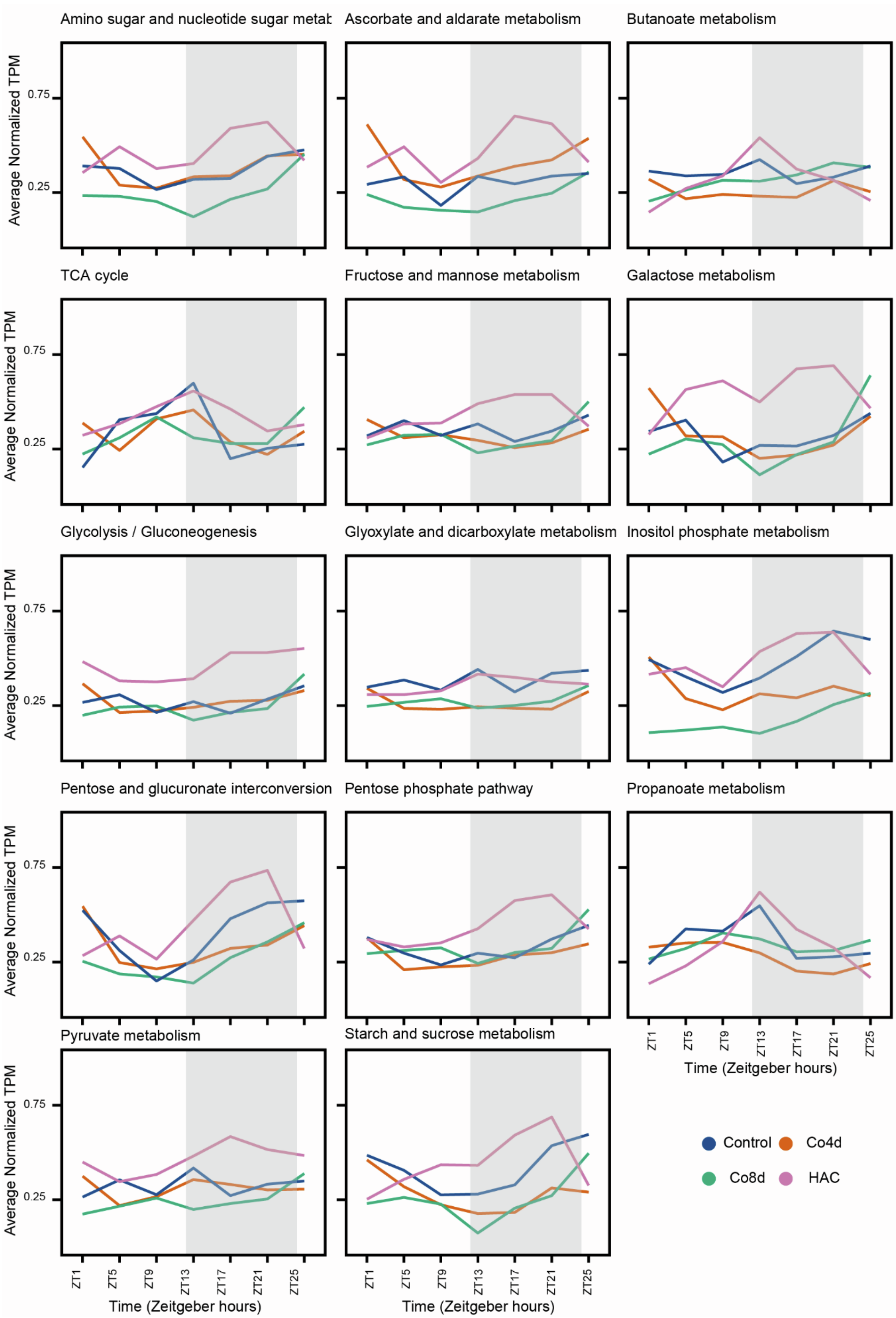

Amino sugar and nucleotide sugar metab
Ascorbate and aldarate metabolism
Butanoate metabolism
TCA cycle
Fructose and mannose metabolism
Galactose metabolism
Glycolysis / Gluconeogenesis
Glyoxylate and dicarboxylate metabolism
Inositol phosphate metabolism
Pentose and glucuronate interconversion
Pentose phosphate pathway
Propanoate metabolism
Pyruvate metabolism
Starch and sucrose metabolism
Average Normalized TPM
0.75
0.25
ZT1
ZT5
ZT9
ZT13
ZT17
ZT21
ZT25
Time (Zeitgeber hours)
Control
Co4d
Co8d
HAC

Figure S10. Temporal expression profiles of pathways related to carbohydrate metabolism. Analysis of time-series transcriptomes showing temporal expression profiles for 14 pathways related to carbohydrate metabolism. Data are shown for four experimental groups: Control, HAC (heterotrophic axenic culture), Co4d (4-day co-culture), and Co8d (8-day co-culture). The pathways include: Amino sugar and nucleotide sugar metabolism; Ascorbate and aldarate metabolism; Butanoate metabolism; TCA cycle; Fructose and mannose metabolism; Galactose metabolism; Glycolysis / Gluconeogenesis; Glyoxylate and dicarboxylate metabolism; Inositol phosphate metabolism; Pentose and glucuronate interconversions; Pentose phosphate pathway; Propanoate metabolism; Pyruvate metabolism; and Starch and sucrose metabolism. Control, axenic culture of *P. tricornutum* on EASW medium; HAC, heterotrophic/autotrophic axenic culture of *P. tricornutum* on co-culture medium; Co4d, 4-day co-culture of *P. tricornutum* with *A. fischeri* on co-culture medium; Co8d, 8-day co-culture of *P. tricornutum* with *A. fischeri* on co-culture medium.

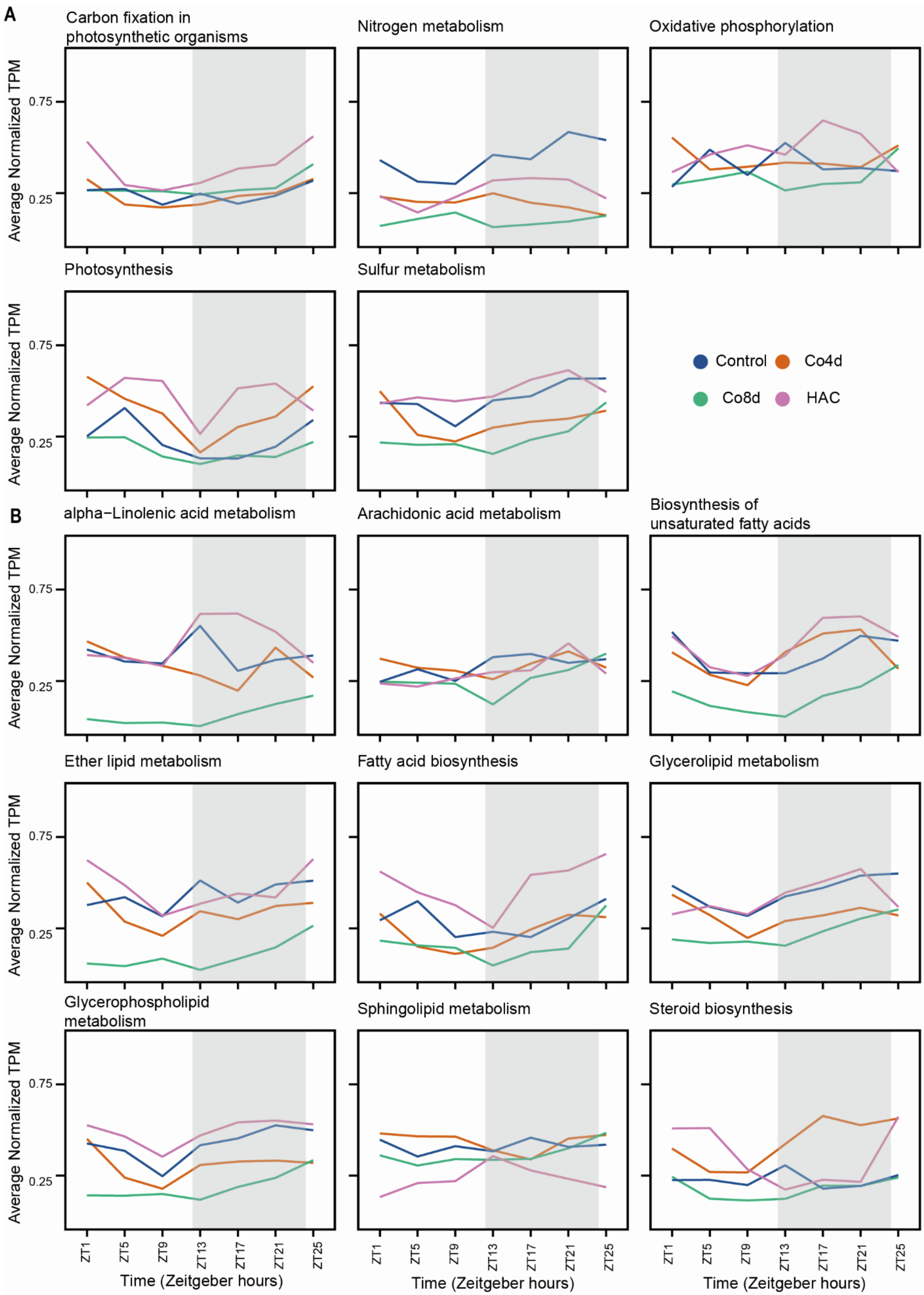

A
Carbon fixation in photosynthetic organisms
Nitrogen metabolism
Oxidative phosphorylation
Photosynthesis
Sulfur metabolism
Control
Co4d
Co8d
HAC
B
alpha−Linolenic acid metabolism
Arachidonic acid metabolism
Biosynthesis of unsaturated fatty acids
Ether lipid metabolism
Fatty acid biosynthesis
Glycerolipid metabolism
Glycerophospholipid metabolism
Sphingolipid metabolism
Steroid biosynthesis
Average Normalized TPM
0.75
0.25
ZT1
ZT5
ZT9
ZT13
ZT17
ZT21
ZT25
Time (Zeitgeber hours)

Figure S11. Temporal expression profiles of pathways related to energy and lipid metabolism. Analysis of time-series transcriptomes across four experimental groups: Control, HAC (heterotrophic axenic culture), Co4d (4-day co-culture), and Co8d (8-day co-culture). (A) Expression profiles for five pathways associated with energy metabolism: Carbon fixation in photosynthetic organisms; Nitrogen metabolism; Oxidative phosphorylation; Photosynthesis; and Sulfur metabolism. (B) Expression profiles for nine pathways associated with lipid metabolism: alpha-Linolenic acid metabolism; Arachidonic acid metabolism; Biosynthesis of unsaturated fatty acids; Ether lipid metabolism; Fatty acid biosynthesis; Glycerolipid metabolism; Glycerophospholipid metabolism; Sphingolipid metabolism; and Steroid biosynthesis. Control, axenic culture of *P. tricornutum* on EASW medium; HAC, heterotrophic/autotrophic axenic culture of *P. tricornutum* on co-culture medium; Co4d, 4-day co-culture of *P. tricornutum* with *A. fischeri* on co-culture medium; Co8d, 8-day co-culture of *P. tricornutum* with *A. fischeri* on co-culture medium.

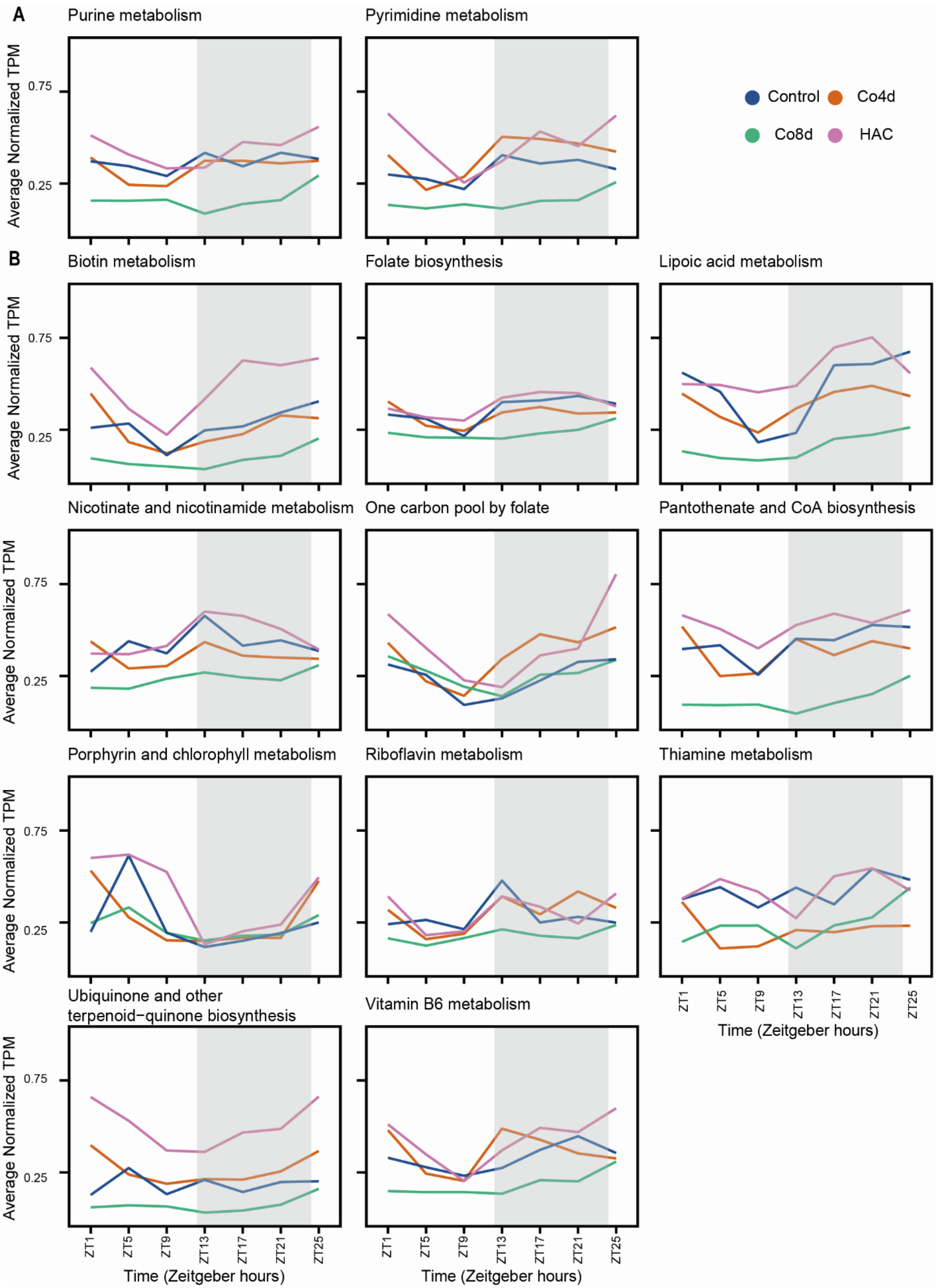

A
Purine metabolism
Pyrimidine metabolism
Control
Co4d
Co8d
HAC
B
Biotin metabolism
Folate biosynthesis
Lipoic acid metabolism
Nicotinate and nicotinamide metabolism
One carbon pool by folate
Pantothenate and CoA biosynthesis
Porphyrin and chlorophyll metabolism
Riboflavin metabolism
Thiamine metabolism
Ubiquinone and other terpenoid−quinone biosynthesis
Vitamin B6 metabolism
Average Normalized TPM
0.75
0.25
ZT1
ZT5
ZT9
ZT13
ZT17
ZT21
ZT25
Time (Zeitgeber hours)

Figure S12. Temporal expression profiles of pathways related to nucleotide metabolism and the metabolism of cofactors and vitamins. Analysis of time-series transcriptomes across four experimental groups: Control, HAC (heterotrophic axenic culture), Co4d (4-day co-culture), and Co8d (8-day co-culture). (A) Expression profiles for two pathways associated with nucleotide metabolism: Purine metabolism and Pyrimidine metabolism. (B) Expression profiles for eleven pathways associated with the metabolism of cofactors and vitamins: Biotin metabolism; Folate biosynthesis; Lipoic acid metabolism; Nicotinate and nicotinamide metabolism; One carbon pool by folate; Pantothenate and CoA biosynthesis; Porphyrin and chlorophyll metabolism; Riboflavin metabolism; Thiamine metabolism; Ubiquinone and other terpenoid-quinone biosynthesis; and Vitamin B6 metabolism. Control, axenic culture of *P. tricornutum* on EASW medium; HAC, heterotrophic/autotrophic axenic culture of *P. tricornutum* on co-culture medium; Co4d, 4-day co-culture of *P. tricornutum* with *A. fischeri* on co-culture medium; Co8d, 8-day co-culture of *P. tricornutum* with *A. fischeri* on co-culture medium.

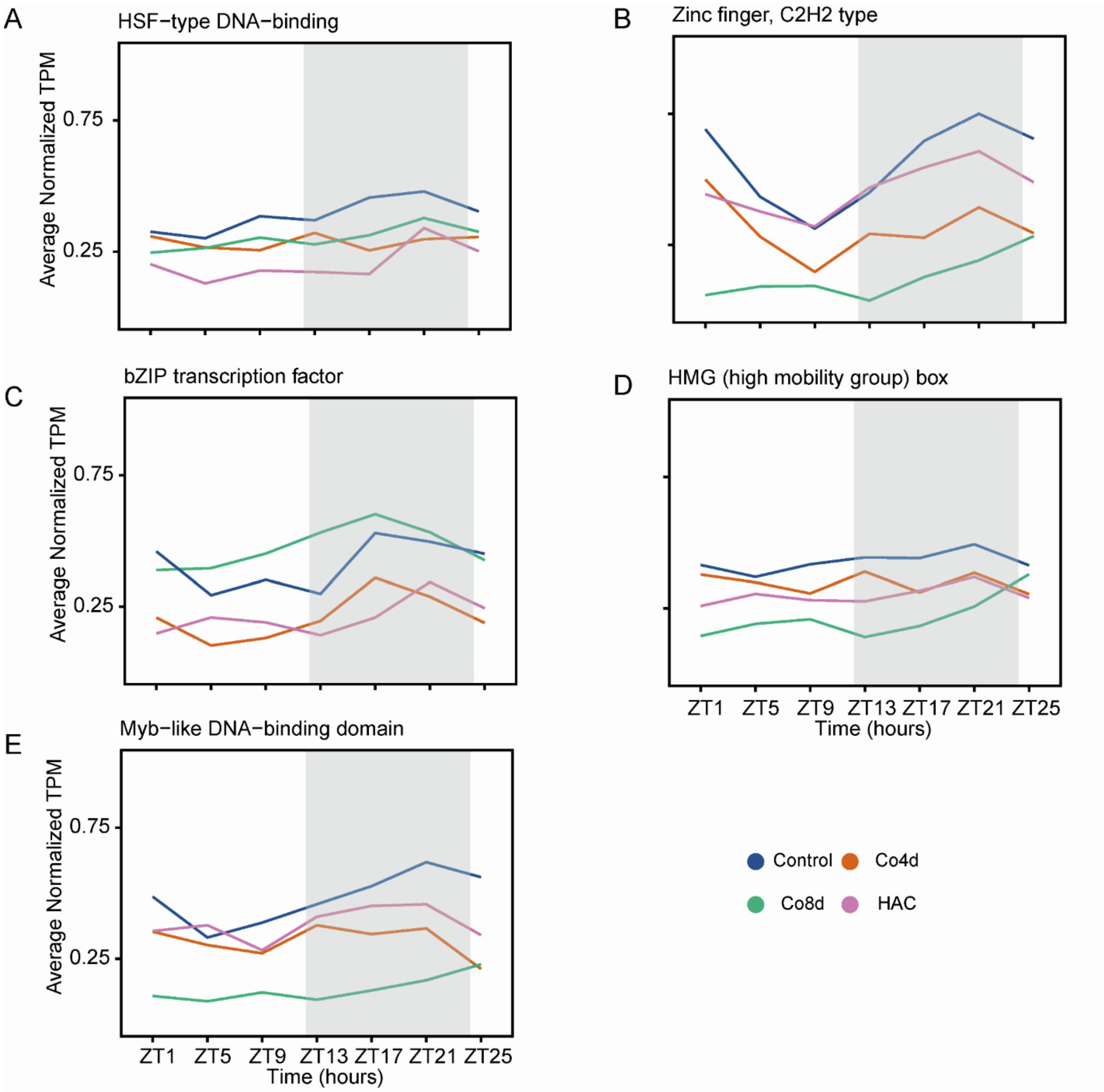

Figure S13. Temporal expression patterns of transcription factors in Control, Co4d, Co8d, and HAC groups. (A) HSF-type DNA-binding domain, (B) Zinc finger, C2H2-type, (C) bZIP transcription factor, (D) HMG (high mobility group) box, (E) Myb-like DNA-binding domain. Transcription factor annotations for the model diatom *Phaeodactylum tricornutum* were retrieved from the JGI PhycoCosm database. Control, axenic culture of *P. tricornutum* on EASW medium; HAC, heterotrophic/autotrophic axenic culture of *P. tricornutum* on co-culture medium; Co4d, 4-

day co-culture of *P. tricornutum* with *A. fischeri* on co-culture medium; Co8d, 8-day co-culture of *P. tricornutum* with *A. fischeri* on co-culture medium.

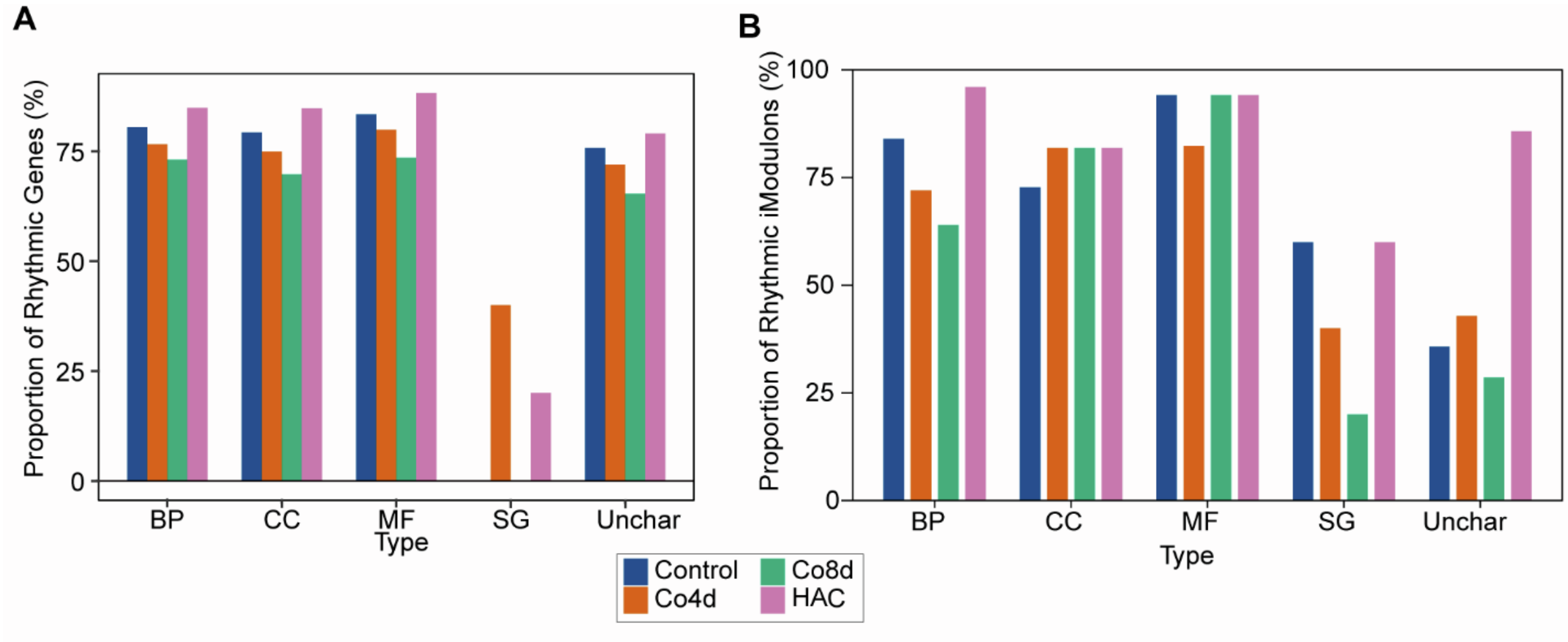

Figure S14. Proportion of rhythmic genes and rhythmic iModulons across functional categories. (A) Proportion of rhythmic genes in each iModulon across functional categories (BP, CC, MF, SG, Unchar), calculated as the percentage of rhythmic genes within each iModulon; (B) Proportion of rhythmic iModulons in each category, determined by applying JTK cycle analysis to iModulon activity profiles. Four experimental conditions are shown: Control, Co4d, Co8d, and HAC. Control, axenic culture of *P. tricornutum* on EASW medium; HAC, heterotrophic/autotrophic axenic culture of *P. tricornutum* on co-culture medium; Co4d, 4-day co-culture of *P. tricornutum* with *A. fischeri* on co-culture medium; Co8d, 8-day co-culture of *P. tricornutum* with *A. fischeri* on co-culture medium.

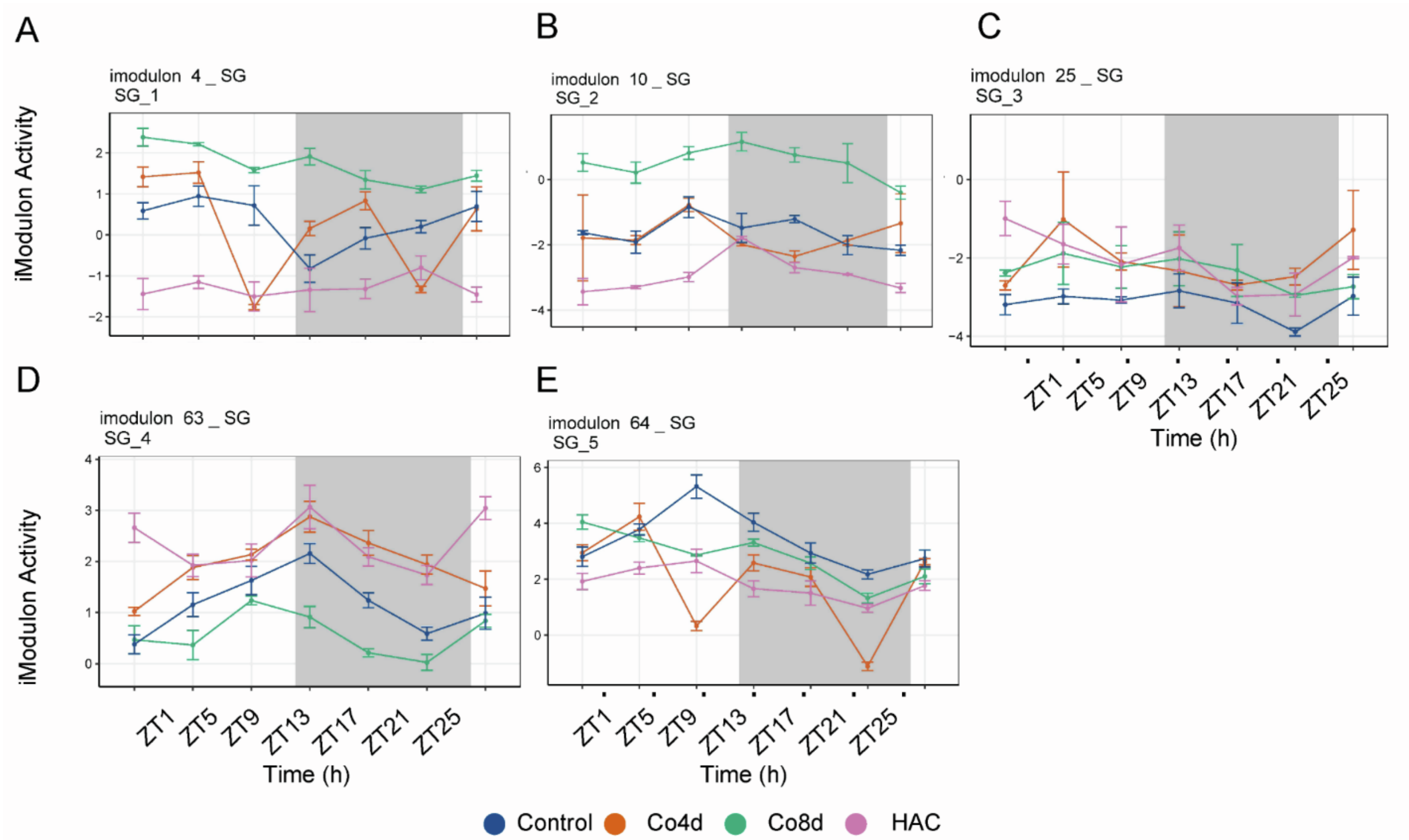

Figure S15. Time-resolved expression patterns of five single-gene iModulons (including SG1 to SG5, where SG denotes single-gene iModulon). Control, axenic culture of *P. tricornutum* on EASW medium; HAC, heterotrophic/autotrophic axenic culture of *P. tricornutum* on co-culture medium; Co4d, 4-day co-culture of *P. tricornutum* with *A. fischeri* on co-culture medium; Co8d, 8-day co-culture of *P. tricornutum* with *A. fischeri* on co-culture medium. Data are presented as mean ± SD from three biological replicates (n = 3), where n represents independent cultures.

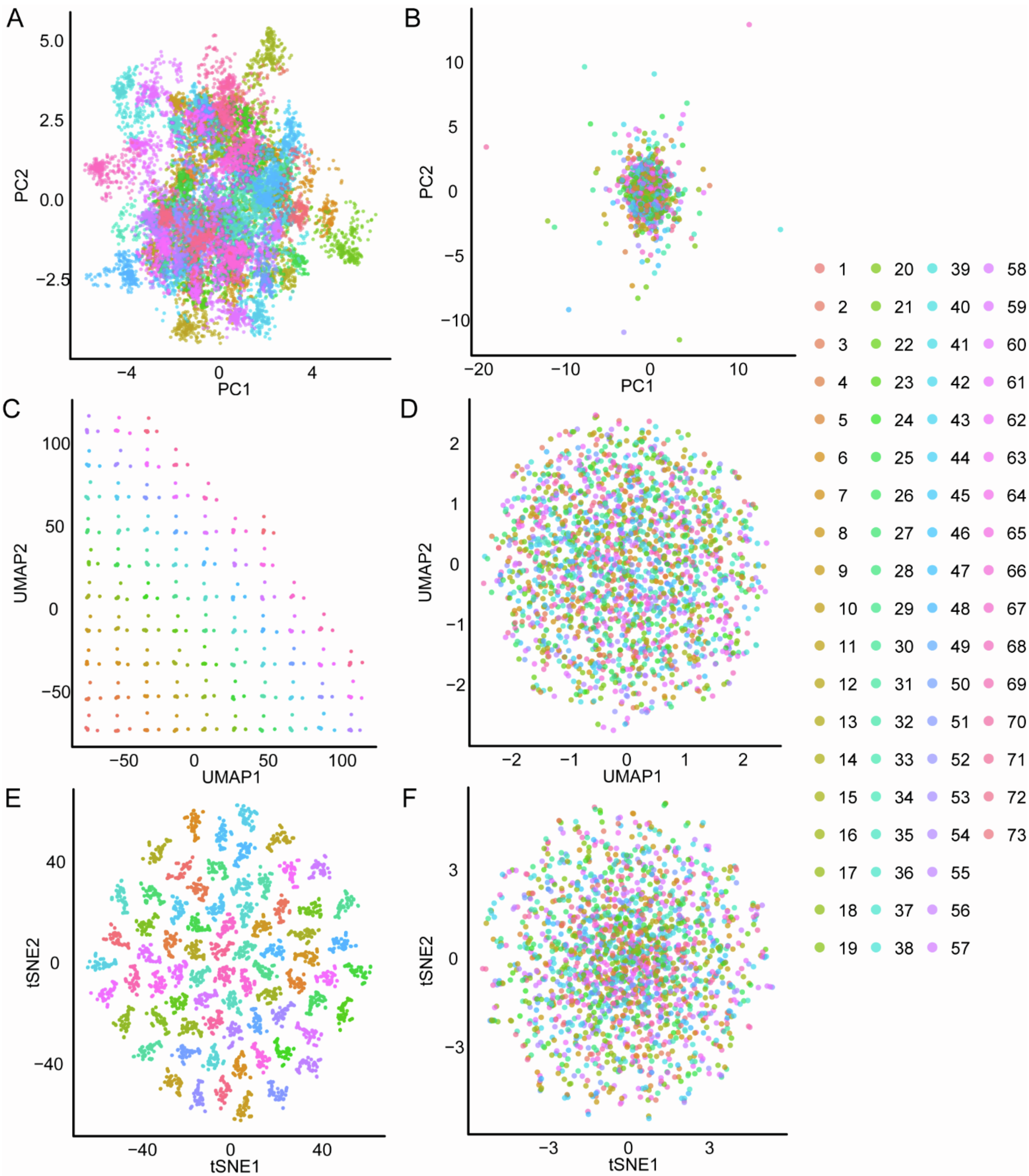

Figure S16. Clustering of FastICA outputs in 130-dimensional space from 73 random runs. (A, C, E) PCA, UMAP, and tSNE visualizations of the A matrix (samples). (B, D, F) PCA, UMAP, and tSNE visualizations of the S matrix (genes). For the M matrix, 2,000 genes were randomly

selected from a total of 10,815 genes for visualization. Dot colors indicate the 73 different FastICA runs.

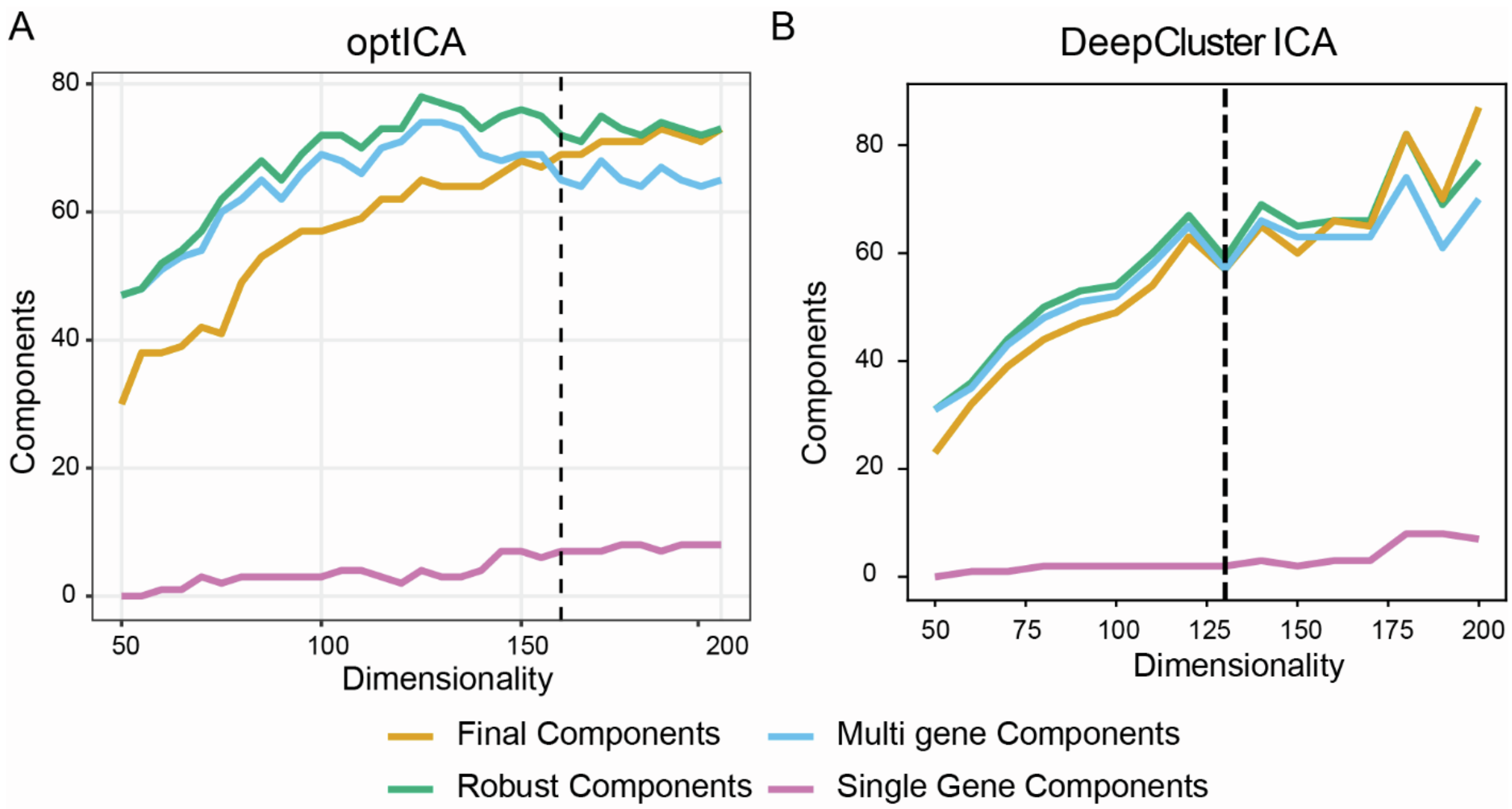

Figure S17. Determination of Optimal IC Dimensionality in optICA (A) and Deepcluster ICA (B).

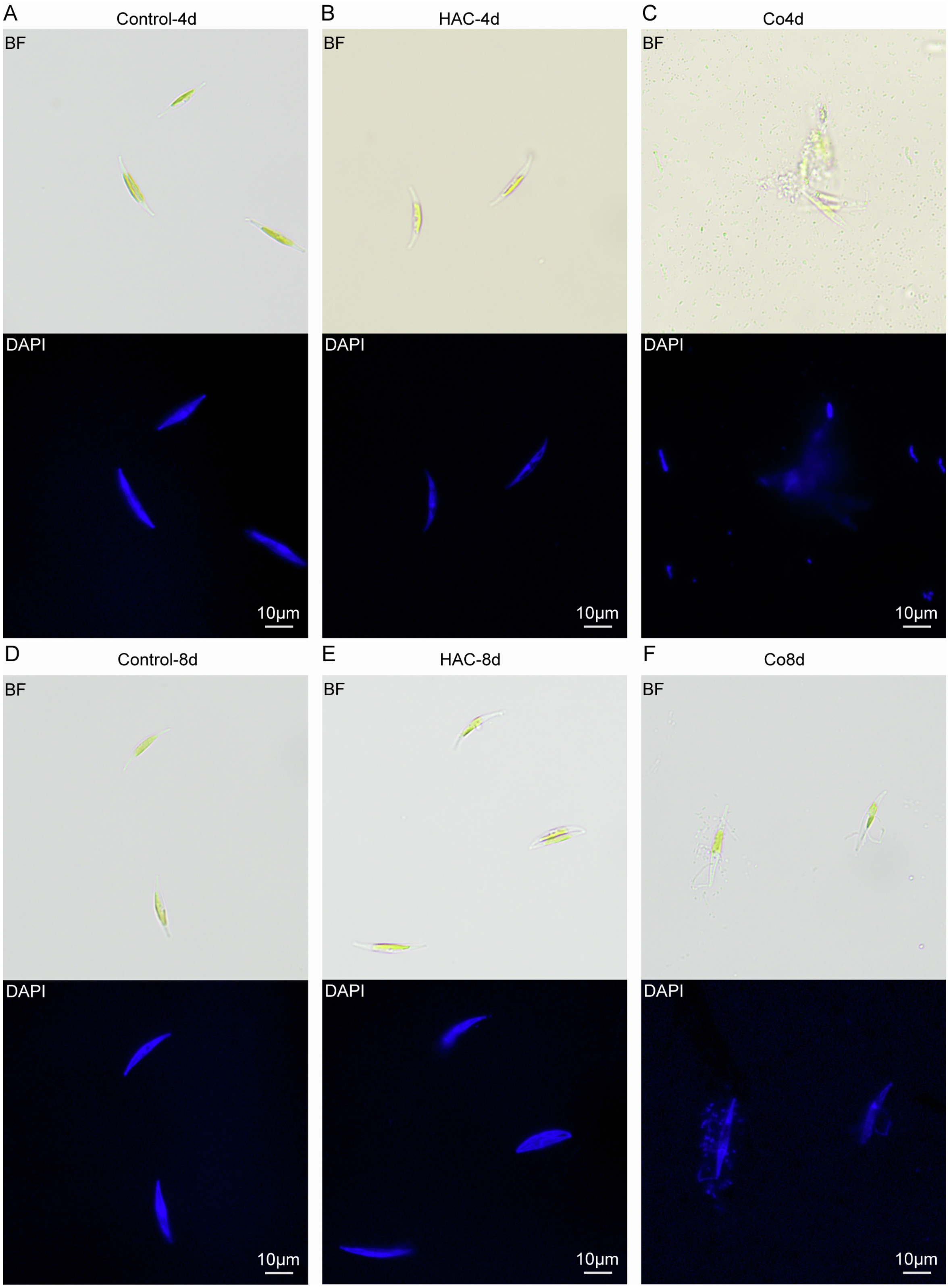
A
Control-4d
BF
DAPI
10μm
B
HAC-4d
BF
DAPI
10μm
C
Co4d
BF
DAPI
10μm
D
Control-8d
BF
DAPI
10μm
E
HAC-8d
BF
DAPI
10μm
F
Co8d
BF
DAPI
10μm

Figure S18. DAPI staining verifies axenic *P. tricornutum* culture and bacterial presence in co-culture. (A) Control-4d; (B) HAC-4d; (C) Co4d; (D) Control-8d; (E) HAC-8d; (F) Co8d. For each panel, bright-field (BF) and corresponding DAPI fluorescence images are shown. Bacterial DAPI fluorescence signals were absent in Control-4d, Control-8d, HAC-4d, and HAC-8d, but present in Co4d and Co8d. Control-4d, axenic culture of *P. tricornutum* on EASW medium for 4 days; Control-8d, axenic culture of *P. tricornutum* on EASW medium for 8 days; HAC-4d, heterotrophic/autotrophic axenic culture of *P. tricornutum* on co-culture medium for 4 days; HAC-8d, heterotrophic/autotrophic axenic culture of *P. tricornutum* on co-culture medium for 8 days; Co4d, 4-day co-culture of *P. tricornutum* with *A. fischeri* on co-culture medium; Co8d, 8-day co-culture of *P. tricornutum* with *A. fischeri* on co-culture medium.

Table S1. Gene information and explained variance of the five single-gene iModulons

iModulon name	Gene name	Explained_variance (%)	Protein
SG_1	Phatr3_J37403	0.014	Senescence-associated protein
SG_2	Phatr3_J41125	0.020	Uncharacterized protein
SG_3	Phatr3_Jdraft998	0.023	Uncharacterized protein
SG_4	Phatr3_EG00893	0.028	NA
SG_5	Phatr3_EG00872	0.044	NA

Table S2. Pseudocode for DeepClusterICA pipeline

Algorithm 1: DeepClusterICA for robust ICA-based module discovery

Input: Expression matrix $X \in \mathbb{R}^{G\times S}$

Output: Module matrix M, mixing matrix A

1. Initialize and pretrain autoencoder AE (encoder dims [G → 512 → 256 → 50], decoder symmetric, epochs=30, lr= 1×10^{-3}, batch size=128)
2. Run repeated ICA for R restarts to obtain N_{vec} components $M_{flat} \in \mathbb{R}^{(Nvec\times G)}$
3. Standardize $\mathbf{M}_{flat}$ column-wise to $\tilde{\mathbf{M}}_{flat}$
4. Encode $\tilde{\mathbf{M}}_{flat}$ using trained AE encoder to latent space $Z \in \mathbb{R}^{(N\_vec\times 50)}$
5. Fit Dirichlet Process Gaussian Mixture Model (DP-GMM) on Z:
6. (K_{max} = 200, covariance type = diag, $\alpha = 1 \times 10^{-15}$, max_iter=1000, random state=0)
7. Assign labels l_i for each component based on DP-GMM
8. Compute repetition ratio R_j for each active cluster j
9. Select robust clusters $C_{robust} = \{ j \mid R_j \geq \tau \}$ (e.g., $\tau = 0.5$)
10. For each $j \in C_{robust}$, compute consensus gene-weight vector $\bar{m}_j$ and sample-activation vector $\bar{a}_j$ by averaging original components assigned to j.
11. Assemble final consensus matrices $\mathbf{M}_{cons}$ and $\mathbf{A}_{cons}$
12. return $\mathbf{M}_{cons}$, $\mathbf{A}_{cons}$

Table S3. Pseudocode for DeepClusterICA pipeline

Component	Parameter	Value
**Autoencoder**	hidden dims	[512, 256]
	latent dim	50
	Epochs	30
	batch size	128
	learning rate	1×10^{-3}
**DP-GMM**	$\boldsymbol{K}_{max}$	200
	A	1×10^{-15}
	covariance type	diag
	max_iter	1000
	random state	0

Table S4. Correspondence between components identified by DeepClusterICA and optICA

DeepCluster ICA_Index	optICA Index	Absolute correlation	DeepCluster ICA Index	optICA Index	Absolute correlation
0	51	0.9950	35	39	0.9394
2	48	0.9616	37	52	0.9940
3	18	0.9696	38	26	0.9735
6	6	0.9753	39	4	0.9927
7	40	0.9647	40	5	0.9533
8	14	0.9842	41	1	0.2321
10	16	0.9367	42	55	0.0883
11	32	0.9850	43	34	0.9276
12	23	0.9883	44	11	0.9942
13	47	0.9500	45	9	0.9781
14	58	0.9804	46	20	0.9760
15	53	0.9419	47	45	0.9846
16	19	0.9877	48	17	0.9870
17	24	0.8678	49	38	0.9929
18	29	0.9351	50	54	0.8928
19	21	0.9865	51	36	0.9629
21	22	0.9894	52	8	0.9961
22	15	0.9506	53	41	0.9783
23	12	0.9866	54	42	0.9947
24	28	0.9574	55	2	0.9824
25	10	0.9613	56	27	0.9703
26	35	0.9880	58	31	0.9590
27	57	0.9655	59	46	0.0838
28	25	0.9829	60	30	0.9770
29	33	0.9796	61	43	0.9831
30	7	0.9745	63	44	0.0978
31	3	0.9921	65	49	0.6962

32	37	0.9765	66	56	0.9554
33	13	0.9901	67	0	0.0696
34	50	0.9523			

Supplementary Methods S1: Detailed methods for DeepCluster ICA and associated supplemental analyses

Running ICA to Identify Robust Components

The analysis pipeline, which encompasses data processing, Independent Component Analysis (ICA), and iModulon characterization, was adapted from the iModulonMiner workflow (https://github.com/SBRG/iModulonMiner) [1-3]. This study included 84 newly generated samples and 1329previously published samples under constant light conditions from our group, totaling 213 samples [4]. After quality control, genes with an average TPM below 2 were excluded, and the expression data were log-transformed into a 10,815 × 213 matrix for ICA analysis.

ICA was performed using the FastICA algorithm from scikit-learn. The algorithm was applied to the log-transformed TPM matrix with 100 iterations, random seeds, and a convergence tolerance of 10^{-7}. Robustness assessment involved clustering iModulons via DBSCAN. To determine the optimal dimensionality for ICA, the analysis was repeated for dimensions ranging from 50 to 200, with increments of 10. The optimal dimension (72) was chosen based on the condition that the number of non-single-gene ICs matched the number of final components (Figure S17).

Each iModulon underwent Gene Ontology (GO) enrichment analysis, with the most significant term (lowest $-\log_{10}p$-value) assigned as its primary annotation. Redundant annotations were differentiated using numerical suffixes. Components were systematically categorized into five classes: uncharacterized modules (Unchar), single-gene iModulons (SG), and modules of Molecular Function (MF), Cellular Component (CC), or Biological Process (BP) based on GO terms.

DeepCluster ICA

Repetitive ICA and Component Extraction. The initial stage of DeepCluster ICA involves a consensus approach to Independent Component Analysis (ICA) on a standardized gene expression matrix [5]. ICA is executed repeatedly with varied random initializations to ensure the stability of the extracted components. From each run, the resulting gene-weight vectors, which define putative gene co-expression modules, are collected. These vectors are then concatenated into a single feature matrix, retaining their run-of-origin metadata [6]. This aggregated matrix forms a robust feature set for all downstream analyses.

Flattening Component Weights and Autoencoder-Based Dimensionality Reduction. Gene-weight vectors from all ICA iterations were concatenated into a single component-by-gene matrix and subsequently standardized (column-wise z-score normalization). To derive a compact feature representation, we trained a shallow autoencoder on this matrix to learn a nonlinear mapping to a low-dimensional latent space [7]. The model was optimized by minimizing the mean squared reconstruction error. The trained encoder was then used to transform each high-dimensional component vector into a dense, low-dimensional embedding, effectively reducing noise and consolidating redundant features across runs for robust clustering.

DP-GMM Clustering in Latent Space. A Dirichlet Process Gaussian Mixture Model (DP-GMM) was employed to perform clustering on the autoencoder-derived latent embeddings [8]. This non-parametric approach automatically determines the optimal number of clusters via variational inference. The model was fit using the Scikit-Learn implementation, with the maximum component number set to 200. Post-convergence, each embedding was assigned a cluster label. The effective number of clusters was defined as the count of Gaussian components with mixing weights greater than 10^{-8}, reflecting the sparsity-inducing nature of the Dirichlet Process prior.

Robust Cluster Selection by Cross-Run Consistency. To ensure the final set of clusters is robust and reproducible, a cross-run consistency filter was applied to the active clusters identified by the DP-GMM. This step is designed to eliminate stochastic artifacts that may arise from specific ICA random initializations. For each active cluster, we calculated a cross-run consistency score, defined as the fraction of total ICA runs that contributed at least one component member to that cluster. Clusters were retained only if their consistency score exceeded a pre-defined threshold (e.g., 0.5), signifying that they were independently identified in at least half of the ICA initializations. The resulting subset, termed "robust clusters," represents stable and consistently detected patterns of gene co-expression, thereby enhancing the reliability of the final biological interpretation.

Consensus Matrix Construction. A representative signature was generated for each robust cluster by computing the consensus of its constituent components. For each cluster, we first collated the original gene-weight and sample-activation vectors corresponding to all components assigned to it. The element-wise mean of these vectors was then calculated to produce a single consensus gene-weight vector and a corresponding consensus sample-activation vector. These consensus vectors were subsequently concatenated column-wise to form the final gene-weight and sample-activation matrices. Each column pair in these matrices defines a robust module (iModulon), representing a stable regulatory signal.

References

1. Rychel, K.; Sastry, A. V.; Palsson, B. O., Machine learning uncovers independently regulated modules in the *Bacillus subtilis* transcriptome. *Nat. Commun.* **2020,** *11* (1).
2. Sastry, A. V.; Gao, Y.; Szubin, R.; Hefner, Y.; Xu, S.; Kim, D.; Choudhary, K. S.; Yang, L.; King, Z. A.; Palsson, B. O., The *Escherichia coli* transcriptome mostly consists of independently regulated modules. *Nat. Commun.* **2019,** *10* (1), 5536.
3. Yuan, Y.; Al Bulushi, T.; Sastry, A. V.; Sancar, C.; Szubin, R.; Golden, S. S.; Palsson, B. O., Machine learning reveals the transcriptional regulatory network and circadian dynamics of *Synechococcus elongatus* PCC 7942. *Proc Natl Acad Sci U S A* **2024,** *121* (38), e2410492121.
4. Su, Y. X.; Hu, J. Y.; Xia, M. S.; Chen, J. W.; Meng, W. Z.; Qian, C.; Shu, Y. X.; Wang, C.; Wang, X. W.; Salehi-Ashtiani, K.; Brynjolfssón, S.; Lin, J. P.; Li, Y. Q.; Zhang, H. S.; Wang, L. Z.; Fu, W. Q., An undiscovered circadian clock to regulate phytoplankton photosynthesis. *Pnas Nexus* **2024,** *3* (11), 14.
5. Hyvärinen, A.; Oja, E., Independent component analysis: algorithms and applications. *Neural Networks* **2000,** *13* (4-5), 411-430.
6. Caron, M.; Bojanowski, P.; Joulin, A.; Douze, M. In *Deep Clustering for Unsupervised Learning of Visual Features*, 15th European Conference on Computer Vision (ECCV), Munich, GERMANY, Sep 08-14 2018; Munich, GERMANY, 2018; pp 139-156.
7. Hinton, G. E.; Salakhutdinov, R. R., Reducing the dimensionality of data with neural networks. *Science* **2006,** *313* (5786), 504-507.
8. Blei, D. M.; Jordan, M. I., Variational Inference for Dirichlet Process Mixtures. *Bayesian Analysis* **2006,** *1* (1), 121-143.
